# STAT3-mediated upregulation of lncRNA HOXD-AS1 as a ceRNA facilitates liver cancer metastasis by regulating SOX4

**DOI:** 10.1186/s12943-017-0680-1

**Published:** 2017-08-14

**Authors:** Hui Wang, Xisong Huo, Xin-Rong Yang, Jia He, Lijun Cheng, Na Wang, Xuan Deng, Haojie Jin, Ning Wang, Cun Wang, Fangyu Zhao, Jingyuan Fang, Ming Yao, Jia Fan, Wenxin Qin

**Affiliations:** 1 State Key Laboratory of Oncogenes and Related Genes, Shanghai Cancer Institute, Renji Hospital, Shanghai Jiao Tong University School of Medicine, No.25/Ln2200 Xie-Tu Road, Shanghai, 200032 China; 2 Department of Liver Surgery, Liver Cancer Institute, Zhongshan Hospital and Key Laboratory of Carcinogenesis and Cancer Invasion of Ministry of Education, Fudan University, Shanghai, 200032 China

**Keywords:** HOXD-AS1, ceRNA, SOX4, Metastasis, Hepatocellular carcinoma

## Abstract

**Background:**

Several of the thousands of human long noncoding RNAs (lncRNAs) have been functionally characterized, yet their potential involvement in hepatocellular carcinoma (HCC) remains poorly understood.

**Methods:**

LncRNA-HOXD-AS1 was identified by microarray and validated by real-time PCR. The clinicopathological significance of HOXD-AS1 was analyzed by Kaplan-Meier method. Chromatin immunoprecipitation was conducted to examine the mechanism of HOXD-AS1 upregulation. The role of HOXD-AS1 in HCC cells was assessed both in vitro and in vivo. ceRNA function of HOXD-AS1 was evaluated by RNA immunoprecipitation and biotin-coupled miRNA pull down assays.

**Results:**

In this study, we found that HOXD-AS1 was significantly upregulated in HCC tissues. Clinical investigation demonstrated high expression level of HOXD-AS1 was associated with poor prognosis and high tumor node metastasis stage of HCC patients, and was an independent risk factor for survival. Moreover, our results revealed that STAT3 could specifically interact with the promoter of HOXD-AS1 and activate HOXD-AS1 transcription. Knockdown of HOXD-AS1 significantly inhibited migration and invasion of HCC cells in vitro and distant lung metastasis in vivo. Additionally, HOXD-AS1 was enriched in the cytoplasm, and shared miRNA response elements with SOX4. Overexpression of HOXD-AS1 competitively bound to miR-130a-3p that prevented SOX4 from miRNA-mediated degradation, thus activated the expression of EZH2 and MMP2 and facilitated HCC metastasis.

**Conclusions:**

In summary, HOXD-AS1 is a prognostic marker for HCC patients and it may play a pro-metastatic role in hepatocarcinogenesis.

**Electronic supplementary material:**

The online version of this article (doi:10.1186/s12943-017-0680-1) contains supplementary material, which is available to authorized users.

## Background

Hepatocellular carcinoma (HCC) is one of the most frequently diagnosed malignancies and the third most common cause of cancer-related death worldwide. Despite the use of innovative therapeutic strategies for HCC, survival rate is still poor for HCC patients. It is mainly due to the high rates of recurrence and metastasis after surgical resection [1, 2]. Although great progress has been achieved in the identification of protein-coding genes or small noncoding RNAs, i.e., microRNAs, in development and progression of HCC [3, 4], little is known about the roles of long noncoding RNAs (lncRNAs) in hepatocarcinogenesis.

LncRNAs are a class of noncoding RNA transcripts that have recently attracted great research interest. So far, a large range of functions have been attributed to lncRNAs, such as control of muscle differentiation [5], reprogramming of induced pluripotent stem cells [6], and modulation of cell apoptosis and invasion [7]. Indeed, accumulating evidence indicates that alteration and dysfunction of lncRNAs have been shown to result in aberrant genes expression that promotes tumor formation, progression and metastasis of many cancer types [8–13]. Several recent reports have described that lncRNAs are important cis- or trans-regulators of genes activities through a variety of mechanisms. In the nucleus, lncRNAs can function as scaffolds to bring proteins to form ribonucleoprotein complexes and as guides to recruit chromatin-modifying complexes to target genes [14–16]. In the cytoplasm, lncRNAs also can function as competing endogenous RNAs (ceRNAs) by competitively binding miRNAs, thereby modulating the derepression of miRNAs targets [17, 18]. To date, only few studies have documented lncRNAs in HCC [19–22] and the underlying mechanisms remain largely unknown.

SOX4, a member of the SOX (SRY-related HMG-box) gene family that containing the highly conserved HMG-domain responsible for specific DNA binding was identified as a common transcription factor that is involved in many cancer progression [23–25]. However, its direct target genes that mediate cancer progression are not well defined.

In the current study, using gene expression profiling analysis we discovered a lncRNA HOXD-AS1 was upregulated in HCC and significantly correlated with poor prognosis of HCC patients. Meanwhile, we demonstrated that HOXD-AS1 promoted HCC metastasis by acting as a ceRNA to upregulate the expression of SOX4 and activated the expression of EZH2 and MMP2, two direct target genes of SOX4.

## Methods

### Cell culture

Human HCC cell lines SNU449, Huh28, SMMC-7721, Huh7, Hep3B, and human embryonic kidney cell line HEK-293 T were cultured in Dulbecco’s Modified Eagle’s Medium (DMEM), supplemented with 10% fetal bovine serum, 100U/ml penicillin, and 100 μg/ml streptomycin. All of cell lines were cultured in a humidified chamber with 5% CO2 at 37 °C. The cells were tested regularly for mycoplasma (R&D Systems’ new MycoProbe Mycoplasma Detection Kit).

### Patients and specimens

Primary human HCC cancerous tissues (C) and corresponding adjacent noncancerous liver tissues (N) were acquired from the surgical specimen archives of Zhongshan Hospital, Shanghai, China. HCC was on the basis of CT, ultrasound or MRI characteristics and biochemistry (AFP serology and liver function enzymes), and was confirmed by histopathology, according to the American Association for the Study of Liver Diseases guidelines [26]. The inclusion criteria were that the samples contained matched tumors (percentage of tumor cells >70%) and corresponding normal liver tissue (>5 cm laterally from the edge of the cancerous region), the patient had a single primary lesion, and no neoadjuvant therapy. The patients who had the history of other solid tumors, had the secondary liver cancer from other primary regions, or did not have the follow-up information were excluded from this study.

Tumor differentiation was graded using the Edmondson grading system. Clinical staging was performed according to the tumor-node-metastasis (TNM) staging system. The follow-up procedures and postoperative treatments were based on a uniform guideline and have been described previously [27]. Overall survival (OS) was calculated from the date of surgery to the date of death. Data were censored at the last follow-up visit or at the time of a patient’s death without relapse. Ethical approval was obtained from the Zhongshan Hospital Research Ethics Committee, and written informed consent was obtained from each patient.

### Microarray analysis

Total RNAs were isolated from the paired tissue samples of 14 HCC patients and purified using TRIzol reagent (Invitrogen, Carlsbad, CA) and RNeasy mini kit (Qiagen Inc, Valencia, CA) according to the manufacturer's protocol. Clinicopathological characteristics of the 14 HCC patients are provided in Additional file 1: Table S1. Following RNA isolation and cDNA synthesis, biotin-labeled cRNA was prepared by Enzo® BioArray^TM^ HighYield^TM^ RNA transcript label kit (Enzo life sciences, INC), then hybridized to the Affymetrix Gene Chip Human Genome U133 Plus 2.0 Array (Santa Clara, CA). Differentially expressed lncRNAs were analyzed as described previously [28].

### 5’and 3’ rapid amplification of cDNA ends (RACE) analyses

5’RACE and 3’RACE analyses were performed with 5 μg of total RNA. The SMARTer™ RACE cDNA kit (Clontech) was used according to the manufacturer’s instructions. The gene specific primers used for PCR are presented in Additional file 2: Table S2.

### Nuclear fractionation

Nuclear fractionation was performed with a PARIS™ Kit (Ambion, Austin, TX). For nuclear fractionation, 1 × 10^7^ cells (Huh7 or SMMC-7721) were collected and resuspended in the cell fraction buffer and incubated on ice for 10 min. After centrifugation, supernatant and nuclear pellet were preserved for RNA extraction using a cell disruption buffer according to the manufacturer’s instructions.

### Fluorescence in situ hybridization analysis

Probe for HOXD-AS1 was presented in Additional file 2: Table S2. SMMC-7721 and Huh7 cells were used for RNA-FISH analysis. Cell suspension was diluted to 100 cells/μL and seeded in the autoclaved glass slides. Slides were treated with 0.2 mol/L HCl for 20 min at room temperature, and washed in 2 × SSC buffer for 5 min. Slides were then incubated in 1%NaSCN for 30 min at 80 °C, and washed in 2 × SSC buffer for 5 min. Slides were incubated in 4% pepsase (2500 ~ 3000U/mg) for 10 min at 37 °C, and washed in 2 × SSC buffer for 5 min, and then fixed in 4% paraformaldehyde for 10 min at room temperature, washed in 2 × SSC buffer for 5 min. Dried the slides and prehybridized with prehybridization buffer for 2 h at 50 °C. Hybridization using HOXD-AS1 probe was performed overnight at 37 °C, and slides were rinsed in 2 × SSC with 0.3%NP-40 (pH7.0 ~ 7.5) for 30 min at 72 °C, washed in 2 × SSC with 0.3%NP-40 for 20 min at room temperature, and counterstained with 4’-6’diamidino-2-phenylindole (DAPI) for 5 min. The images were acquired using a confocal microscope (Leica).

### RNA immunoprecipitation

RNA immunoprecipitation (RIP) experiments were performed with a Magna RIP™ RNA-Binding Protein Immunoprecipitation Kit (Millipore, Billerica, MA) according to the manufacturer’s instructions. AGO2 antibody was used for RIP (Cell Signaling Technology, Beverly, MA). Co-precipitated RNAs were detected by real-time PCR. Total RNAs (input controls) and IgG controls were assayed simultaneously to demonstrate that the detected signals were the result of RNAs specifically binding to AGO2. The gene-specific primers used for detecting HOXD-AS1 and SOX4 were presented in Additional file 2: Table S2.

### Chromatin immunoprecipitation

A chromatin immunoprecipitation (ChIP) assay was performed using the EZ-ChIP^TM^ kit (Millipore, Billerica, MA) according to the manufacturer’s instructions. The following antibodies were utilized to immunoprecipitate crosslinked protein-DNA complexes: rabbit anti-STAT3 (10253-2-AP, Protein tech), rabbit anti-SOX4 (ARP38234, AVIVA) and normal rabbit IgG (12–370, Millipore). The immunoprecipitated DNA was purified for quantitative PCR analyses with specific primers. The primers were listed in Additional file 2: Table S2.

### Pull down of biotin-coupled miRNA

Biotin was attached to the 3’-end of miR-130a-3p or negative control mimics. 1 × 10^6^ HCC cells were transfected with 100 pmol Bi-miR-130a-3p, or negative control using Lipofectamine 2000 according to the manufacturer’s protocol. Forty-eight hours later, cells were pelleted at 1000 rpm. After washing twice with PBS, cell pellets were resuspended in 0.7 ml lysis buffer (5 mM MgCl_2_, 100 mM KCl, 20 mM Tris (pH7.5), 0.3% NP-40, 50U of RNase OUT (Invitrogen, USA)), complete protease inhibitor cocktail (Roche Applied Science, IN), and incubated on ice for 10 min. The cell lysate was isolated by centrifugation at 10,000 g for 10 min. miRNA biotin pull down experiments were performed according to previous reports [29]. The level of HOXD-AS1 or SOX4 in the pull down of Biotin-miR-130a-3p or negative control was quantified by real-time PCR.

### Oligonucleotide transfection

Small interfering (si) RNA duplexes were designed and synthesized by Genepharma (Shanghai, China). MiR-130a-3p mimics, inhibitors and corresponding negative control (NC) were synthesized by Ribobio (Guangzhou, China). The sequences used are shown in Additional file 2: Table S2. Cells were transfected using Lipofectamine 2000.

### Real-time PCR

Total RNA was extracted from cells using Trizol reagent (Invitrogen) according to the manufacturer’s instruction. A total of 1 μg of RNA was subjected to reverse transcription using HiScript II Q RT SuperMix for qPCR (+gDNA wiper) (Vazyme, Nanjing, China) and real-time PCR was carried out using AceQ qPCR SYBR Green Master Mix (Vazyme, Nanjing, China). The PCR reaction conditions were as follows: 95 °C for 15 s followed by 40 cycles of 95 °C for 5 s and 60 °C for 30s. The expression levels were normalized against those of the internal reference gene β-actin, the relative expression levels were determined by the following equation: 2-ΔΔCt (ΔCt = ΔCttarget - ΔCtβ-actin). A list of primers used for real-time PCR experiments were in Additional file 2: Table S2.

### Western blot analysis

Proteins from cell lysates were prepared in 1 × sodium dodecyl sulfate buffer, separated by sodium dodecyl sulfate–polyacrylamide gel electrophoresis (SDS-PAGE) and transferred to a nitrocellulose membrane (Bio-Rad, Hercules, CA, USA). The membranes were blocked with 5% non-fat milk and incubated with the appropriate antibody. Antigen-antibody complex was detected with enhanced chemiluminescence reagents (Pierce, Rockford, IL). Antibodies used in this study were shown in Additional file 3: Table S3.

### In vitro assays for migration and invasion

For transwell migration assay, 5 × 10^4^ cells were plated in the top chamber of each insert (BD Biosciences, NJ, USA) with a non-coated membrane. For invasion assay, 8 × 10^4^ cells were placed in the upper chamber of each Matrigel-coated insert (BD Biosciences, Billerica, MA, USA). After 18 h of incubation at 37 °C, cells that migrated or invaded were fixed and stained in dye solution containing 0.1% crystal violet and 20% methanol. The number of cells that had migrated or invaded was counted and imaged using an IX71 inverted microscope (Olympus Corp, Tokyo, Japan).

### In vivo assay for metastasis

For in vivo metastasis assays, 3 × 10^6^ Huh7 cells infected with shRNA-HOXD-AS1 or empty vector were suspended in 300 μL of serum-free DMEM per male BALB/c-nu/nu mice, and injected into nude mice through the tail vein (12 mice per group). After six weeks, mice were sacrificed, and their lungs were dissected, fixed with phosphate-buffered neutral formalin, and prepared for standard histological examination. Mice were handled and housed according to protocols approved by the Shanghai Medical Experimental Animal Care Commission.

### Luciferase reporter assay

HEK-293 T cells were seeded in 96-well plates at a density of 5000 cells per well. After 24 h, the cells were transiently transfected with a mixture of 5 ng of pRL-CMV Renilla luciferase reporter, 50 ng of the firefly luciferase reporter, and 5pmol small RNA (siRNA or miRNA mimics). After 48 h, luciferase activity was measured using the dual-luciferase reporter assay system (Promega, Madison, WI, USA).

### Statistical analysis

Results are presented as the means ± SD. Data were analyzed using Student’s *t*-test (two-tailed, with *p* < 0.05 considered significant) unless otherwise specified (paired *t*-test, *χ*2 test or Spearman correlation). Cumulative survival was evaluated using the Kaplan-Meier method (log-rank test). All the statistical analyses were performed using the SPSS 16.0 software (SPSS, Inc., Chicago, IL).

## Results

### HOXD-AS1 is upregulated and positively associated with poor prognosis in HCC patients

**Fig. 1:**
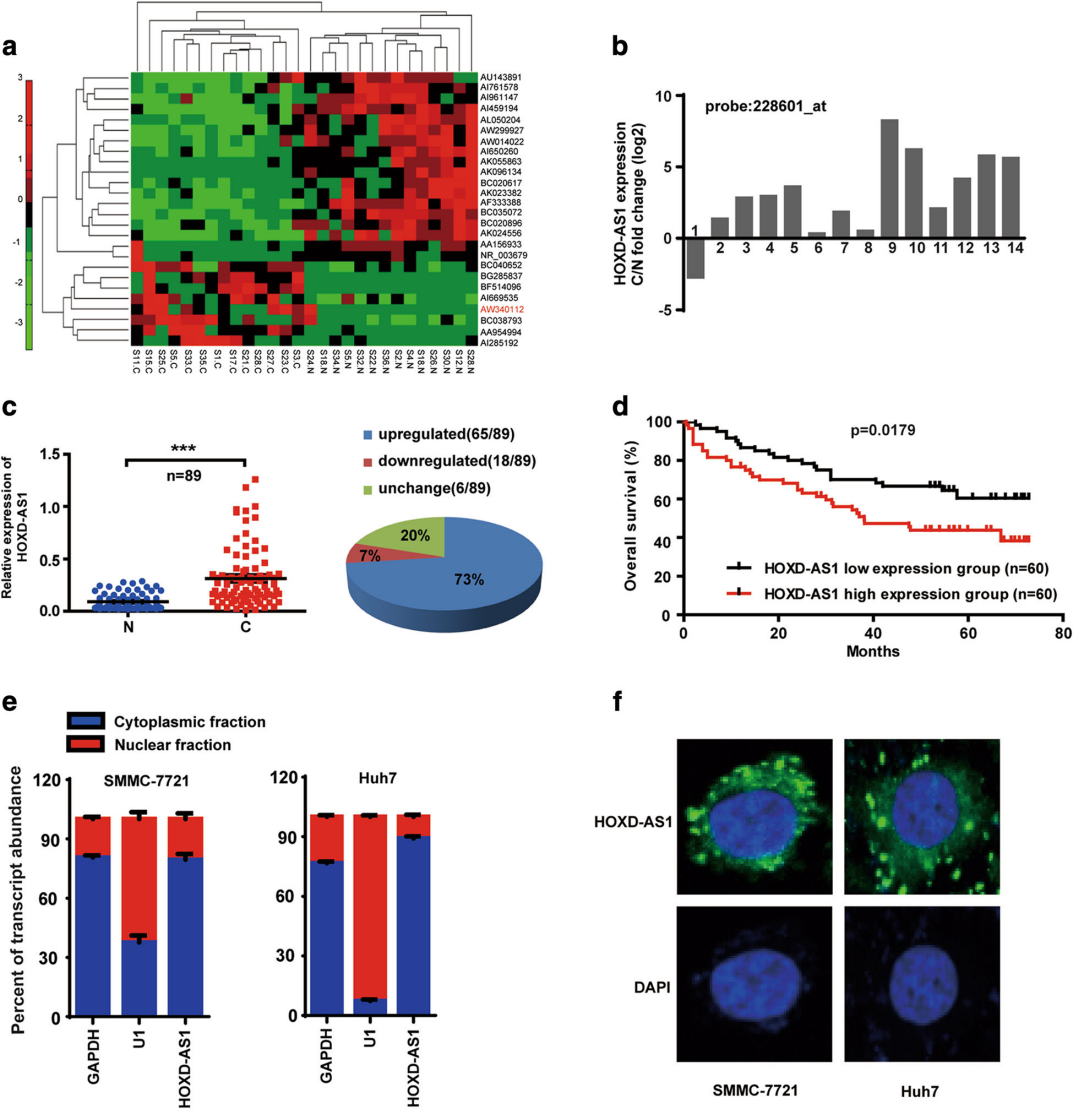
HOXD-AS1 is upregulated and positively associated with poor prognosis in HCC patients. **a** Hierarchical clustering analysis of 26 lncRNAs that were differentially expressed in 14 paired samples of cancerous tissues (**c**) and corresponding adjacent noncancerous liver tissues (N) (fold change > 2^2^ or <2^-2^, *p* < 0.05). Expression values are represented in shades of *red* and *green*. **b** The fold change of HOXD-AS1 expression in 14 HCC tissues (probe: 228601_at). **c** Left: HOXD-AS1 expression in 89 paired samples of HCC tissues (*p* < 0.0001). Right: Pie chart of the proportions of HCC samples in which HOXD-AS1 expression was upregulated (*blue*), downregulated (*red*), or showed no change (*green*). **d** Correlation between HOXD-AS1 expression and overall survival in 120 HCC patients. Median value of HOXD-AS1 expression was chosen as the point for separating tumors with low or high-level expression group. (Log-rank; *p* = 0.0179). **e** The expression levels of HOXD-AS1 in nuclear and cytoplasm of HCC cells. U1 (nuclear retained) and GAPDH (exported to cytoplasm) were used as controls. **f** Fluorescence in situ hybridization analysis of the subcellular location of HOXD-AS1 in HCC cells

To uncover the potential involvement of lncRNAs in HCC, we performed global gene expression analysis for total RNAs from the paired samples of cancerous tissues (C) and corresponding adjacent noncancerous liver tissues (N) of 14 HCC patients with Affymetrix GeneChip Human Genome U133 Plus 2.0 Array (Santa Clara, CA) (GEO Submission: GSE84402) [30]. In total, we identified 26 lncRNAs that were significantly differentially expressed (fold change >2^2^ or <2^-2^, *p* < 0.05) (Fig. 1a, Additional file 4: Table S4). In all 26 dysregulated lncRNAs, AW340112 (termed HOXD-AS1) was most upregulated in HCC tissues (fold change >8, Additional file 4: Table S4), it was expressed at a significantly high level in most of HCC tissues according to probe signal calculation (Fig. 1b, Additional file 5: Figure S1). We next assessed the expression of HOXD-AS1 in 89 paired samples of HCC tissues and corresponding adjacent noncancerous liver tissues. The results showed that HOXD-AS1 was significantly increased in HCC tissues, the upregulated expression of HOXD-AS1 was observed in 73% (65/89) of HCC (*p* < 0.0001) (Fig. 1c).

To further investigated the clinicopathological and prognostic significance of HOXD-AS1 levels in HCC patients, 120 HCC tissues (all of which had detailed clinical and follow-up data available) were examined. Our results showed that upregulation of HOXD-AS1 was significant correlated with TNM stage in HCC patients (Table 1). Furthermore, Kaplan-Meier analysis revealed that high level of HOXD-AS1 was associated with poor overall survival (OS) in HCC patients (Fig. 1d; *p* = 0.0179). Multivariate regression revealed that the expression level of HOXD-AS1 was an independent and significant factor for OS (HR: 0.552; 95% CI: 0.321–0.950; *p* = 0.032) (Table 2).

**Table 1:** Correlation between the expression levels of HOXD-AS1 and clinicopathological features in 120 HCC patients

	HOXD-AS1		*p*-value
	Low	High	
All cases	60	60	
Sex			
Male	15	8	0.104
female	45	52	
Age			
≤ 55	36	35	0.853
> 55	24	25	
Liver cirrhosis			
Yes	51	53	0.591
No	9	7	
AFP(ng/mL)			
≤ 20	26	24	0.711
> 20	34	36	
Tumor size (cm)			
≤ 5	37	33	0.459
> 5	23	27	
Tumor number			
Single	55	50	0.269
Multiple	5	10	
Tumor thrombus			
Single	41	35	0.344
Multiple	19	25	
Encapsulation			
Complete	25	22	0.575
No	35	38	
TNM stage			
I	43	38	*0.042* ^a^
II	17	16	
III	0	6	

*Abbreviations*: *HCC* hepatocellular carcinoma, *AFP* α-fetoprotein, *TNM* tumor-nodes-metastases, ^a^statistically significant

**Table 2:** Multivariate analyses of factors associated with survival in 120 HCC patients

		Multivariate		
Variable	Univariate p	HR	95% CI	*p*-value
Sex: male vs female	0.430			NA
Age: ≤55 vs >55	0.967			NA
Serum AFP: ≤20 ng/ml vs >20 ng/ml	0.098			NA
Liver cirrhosis: no vs yes	0.568			NA
TNM: I vs II vs III	0.146			NA
Tumor encapsulation: no vs complete	*0.046* ^a^			NS
Tumor size: ≤5 cm vs >5 cm	*<0.0001* ^a^	0.376	0.213–0.662	*0.001* ^a^
Tumor number: single vs multiple	*0.003* ^a^	0.492	0.256–0.945	*0.033* ^a^
Tumor thrombus: single vs multiple	*<0.0001* ^a^	0.348	0.197–0.612	*<0.0001* ^a^
HOXD-AS1: low vs high	*0.018* ^a^	0.552	0.321–0.950	*0.032* ^a^

*Abbreviations*: *HCC* hepatocellular carcinoma, *HBsAg* hepatitis B surface antigen, *AFP* α-fetoprotein, *TNM* tumor-nodes-metastases, *HR* hazard ratio, *95% CI* 95% confidential interval, *NA* not applicable, *NS* not significant, ^a^statistically significant

HOXD-AS1 is transcribed in antisense orientation of the protein-coding genes HOXD1. We obtained the full-length sequence of HOXD-AS1 by RACE analyses, it is composed of 3956 nucleotides and consists of four exons and three introns with 3’ polyadenylate tail (Additional file 6: Figure S2). Furthermore, subcellular fractionation analysis showed that HOXD-AS1 was predominantly abundant in the cytoplasm of HCC cells (Fig. 1e). In situ hybridization analysis also showed that HOXD-AS1 was located mainly in the cytoplasm (Fig. 1f).

### HOXD-AS1 is regulated by the transcription factor STAT3

**Fig. 2:**
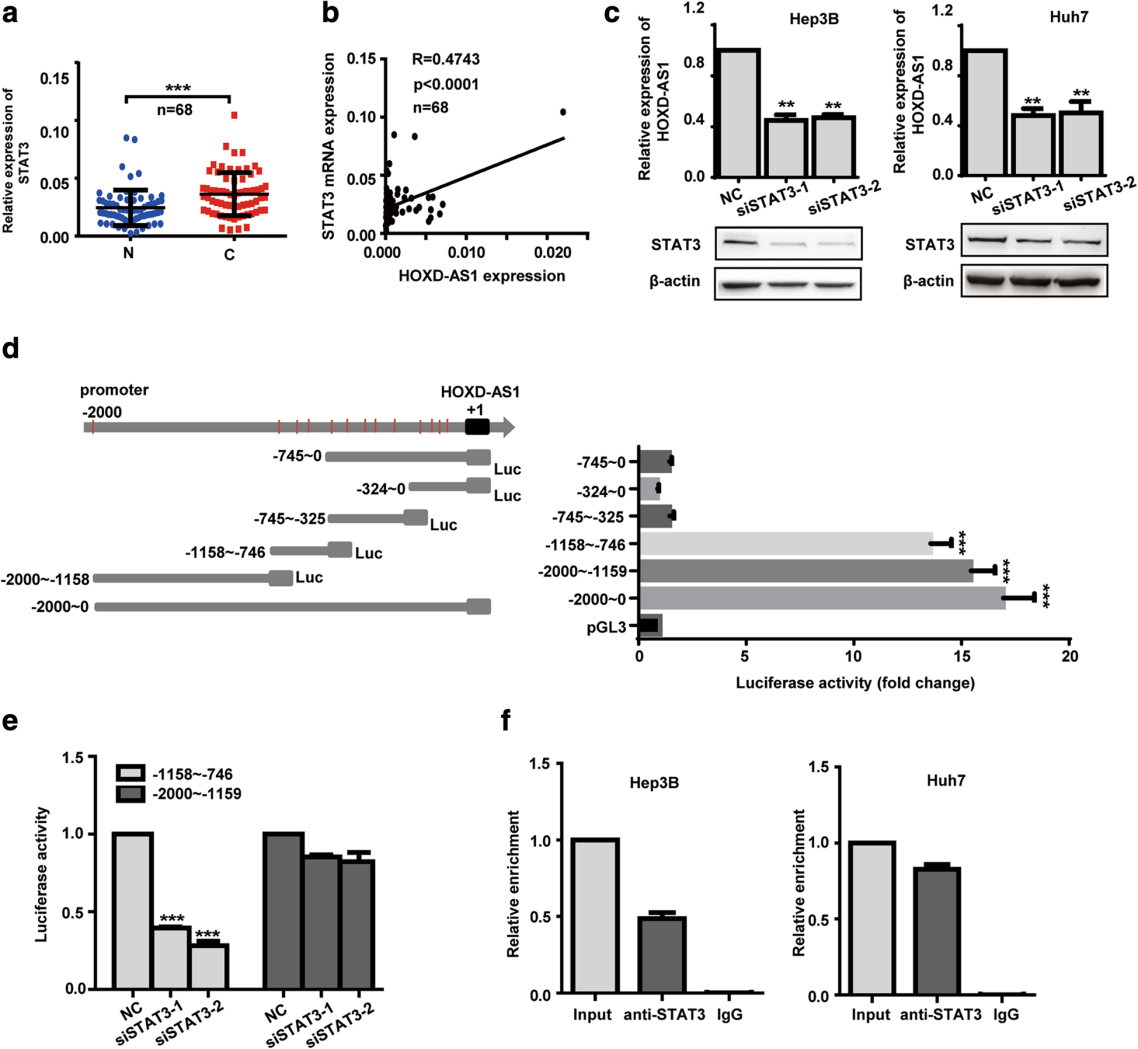
HOXD-AS1 is regulated by the transcription factor STAT3. **a** mRNA expression level of STAT3 in 68 paired samples of HCC tissues. **b** Expression correlation analysis between HOXD-AS1 (x) and STAT3 (y) in 68 HCC tissues (R = 0.4743, *p* < 0.0001). **c** Expression levels of HOXD-AS1 in Hep3B and Huh7 cells after knockdown of STAT3. **d** Left: schematic representation of constructs for HOXD-AS1 promoter reporter. Right: luciferase activities of 6 truncated constructs in HEK-293 T cells. **e** Luciferase activities of -1158 bp ~ -746 bp or -2000 bp ~ -1159 bp fragments after knockdown of STAT3. **f** ChIP assay in HCC cells, followed by quantitative PCR amplification of binding site 2 within HOXD-AS1 promoter region. Genomic DNA input was 1%. Data in (**a**-**e**) are the means ± SD. **p < 0.01, and ***p < 0.001

To explore the mechanism of HOXD-AS1 upregulation in HCC, we applied online bioinformatical software programs JASPAR (http://jaspar.genereg.net/cgi-bin/jaspar_db.pl) to analyze promoter region of HOXD-AS1 and 13 potential sites of STAT3 binding were found. Next, we examined the mRNA expression level of STAT3 in 68 HCC specimens. We found that expression level of STAT3 was significantly upregulated, and was positively correlated with HOXD-AS1 in HCC tissues (Fig. 2a-b). To further investigate the correlation between HOXD-AS1 and STAT3, we detected the transcriptional level of HOXD-AS1 and the protein level of phosphorylated STAT3 in the same group of HCC tissues. We found that active STAT3 was also positively correlated with HOXD-AS1 in 30 HCC tissues (Additional file 7: Figure S3A-B). Then, we measured mRNA levels of HOXD-AS1 in the case of STAT3 knockdown. The results revealed that STAT3 knockdown led to a significant decrease of HOXD-AS1 in Hep3B and Huh7 cells (Fig. 2d).

To confirm HOXD-AS1 was a transcriptional target of STAT3, serial truncations of HOXD-AS1 promoter were cloned into pGL3-basic vector, and luciferase activity was measured after transfection of these constructs into HEK-293 T cells. The highest activities were associated with -2000 nt ~ -1159 nt and -1158 nt ~ -746 nt (Fig. 2e), indicating that these two fragments contained regulatory elements and they were critical for the transcription of HOXD-AS1. Next, we cotransfected luciferase reporter and siRNA against STAT3 into HEK-293 T cells. The results showed that STAT3 knockdown significantly reduced luciferase activity of -1158 nt ~ -746 nt fragment, however, luciferase activity of reporter containing -2000 nt ~ -1159 nt was not affected (Fig. 2f). These results indicate that the region between -1158 bp to -746 bp on the HOXD-AS1 promoter is responsible for STAT3-mediated activation of HOXD-AS1. Sequence analysis of -1158 nt ~ -746 nt fragment uncovered three putative STAT3 binding sites, located at -828 nt ~ -818 nt (site 1), -938 nt ~ -928 nt (site 2), and -1121 nt ~ -1111 nt (site 3). To determine which STAT3 binding sites were responsive to STAT3-mediated transcriptional activation of HOXD-AS1. ChIP assay was performed. Our results showed that STAT3 could directly bind to the site 2 on the HOXD-AS1 promoter in HCC cell lines (Fig. 2g). These findings indicate that the upregulation of HOXD-AS1 is mediated by STAT3 in HCC.

### HOXD-AS1 promotes invasion and metastasis of HCC cells in vitro and in vivo

**Fig. 3:**
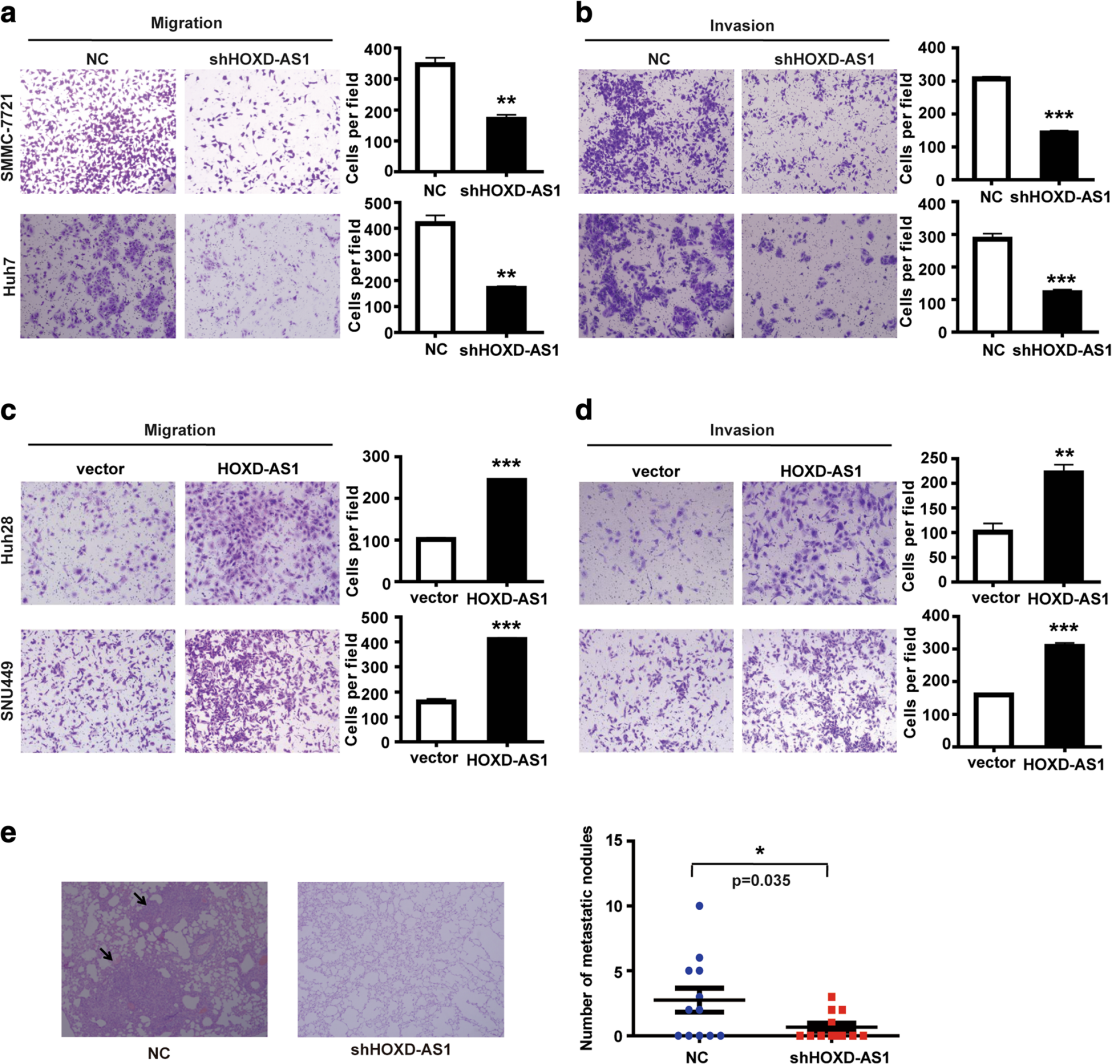
HOXD-AS1 promotes invasion and metastasis of HCC cells in vitro and in vivo*.* **a, b** Migration and invasion analyses of SMCC-7721 and Huh7 cells with shRNA knockdown of HOXD-AS1. Representative images are shown on the left, and the average number of cells per field at the indicated time points is shown on the right. **c, d** Migration and invasion assays of Huh28 and SNU449 cells were performed after transduction with HOXD-AS1 or control lentivirus, respectively. **e** Gross morphology of representative lungs and characteristic H&E staining of metastatic nodules in the lung of nude mice 6 weeks after tail vein injection with tumor cells. The numbers of metastatic nodules in the lungs of each mouse (*n* = 12 mice per group) were counted and analyzed using. Data in A-E are the means ± SD. *p < 0.05, **p < 0.01, and ***p < 0.001

To investigate the function of HOXD-AS1 on cell biological behavior, we first determined the expression levels of HOXD-AS1 in various HCC cell lines. The results showed that HOXD-AS1 expression was relatively high in SMMC-7721 and Huh7 cells, whereas it was relatively low in Huh28 and SNU449 cells (Additional file 8: Figure S4A). We therefore constructed a lentivirus vector harboring shRNA-HOXD-AS1 and established two stable knockdown cell lines in SMMC-7721 and Huh7 (Additional file 8: Figure S4B). Meanwhile, we stably overexpressed HOXD-AS1 in SNU449 and Huh28 cell lines (Additional file 8: Figure S4C). Transwell assays showed that migratory and invasive abilities of SMMC-7721 and Huh7 cells were significantly reduced when HOXD-AS1 was decreased (Fig. 3a-b). In contrast, exogenous expression of HOXD-AS1 dramatically promoted migration and invasion in Huh28 and SNU449 cells (Fig. 3c-d). However, HOXD-AS1 did not affect HCC cell proliferation and apoptosis (data not shown). To explore the effect of HOXD-AS1 on tumor metastasis in vivo, we implanted either control or Huh7 cells with stable HOXD-AS1 knockdown into nude mice via the tail vein. The results showed that HOXD-AS1 knockdown significantly decreased the number of metastatic lung nodules compared with the control cells (*n* = 12, *p* = 0.035) (Fig. 3e).

### HOXD-AS1 is involved in migration of HCC cells through regulating SOX4

**Fig. 4:**
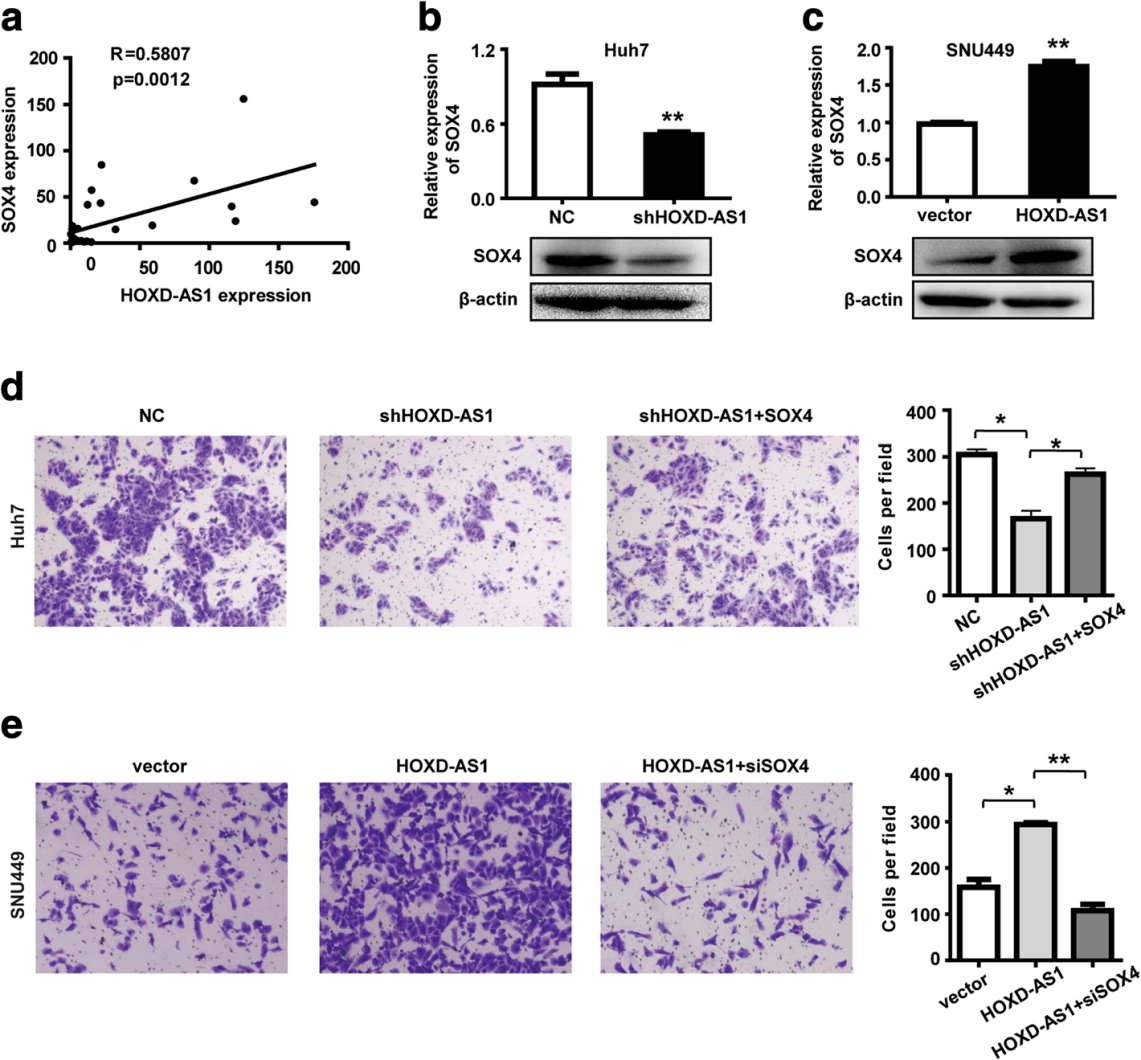
HOXD-AS1 is involved in migration of HCC cells through regulating SOX4. **a** Expression correlation between HOXD-AS1 (x) and SOX4 (y) in 14 HCC tissues. Data are depicted as probe signal value of global gene expression microarray (R = 0.5807, *p* = 0.0012). **b, c** The expression of SOX4 in Huh7 cells with HOXD-AS1 knockdown and SNU449 cells with HOXD-AS1 overexpression. β-actin was used as the loading control. **d** Migration assays of Huh7 cells with HOXD-AS1 knockdown and SOX4 overexpression. **e** Migration assays of SNU449 cells with HOXD-AS1 overexpression and SOX4 knockdown. Data in **b**-**e** are the means ± SD. *p < 0.05 and **p < 0.01

To identify the mechanism of HOXD-AS1 in HCC cell metastasis, we analyzed the co-expression patterns of HOXD-AS1 and core transcript factors (TFs) in the 14 HCC tissues used in global gene expression analysis. Five TFs were highly correlated with the level of HOXD-AS1, which are SOX4, E2F7, E2F8, NFYA, and TCF19 (Fig. 4a, Additional file 9: Figure S5A). We therefore hypothesized that HOXD-AS1 might regulate these TFs in HCC cells. To test this hypothesis, we first investigated whether HOXD-AS1 knockdown affected expression levels of these five TFs. Our results showed that the expression level of SOX4 was downregulated after HOXD-AS1 reduction in Huh7 cells (Fig. 4b), however, expression levels of E2F7, E2F8, NFYA, and TCF19 were not affected significantly (Additional file 9: Figure S5B). We then examined levels of five TFs in SNU449 cells with HOXD-AS1 overexpression. As a result, we found that only SOX4 significantly upregulated when HOXD-AS1 was ectopically overexpressed in SNU449 cells (Fig. 4c, Additional file 9: Figure S5C). To further determine functional association between HOXD-AS1 and SOX4, cell migration after overexpression or knockdown of SOX4 was examined. We found that the suppression of migratory ability by HOXD-AS1 knockdown was reversed when simultaneously overexpressing SOX4 in Huh7 cells (Fig. 4d). In addition, knockdown of SOX4 abrogated the effects of HOXD-AS1 overexpression on SNU449 cell migration (Fig. 4e). These results indicate that SOX4 is necessary for HOXD-AS1 to regulate HCC metastasis.

### HOXD-AS1 regulates SOX4 expression through a miRNA-dependent mechanism

**Fig. 5:**
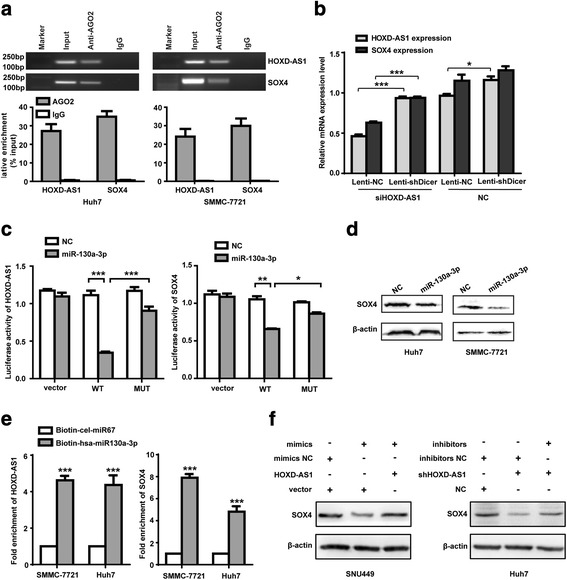
HOXD-AS1 regulates SOX4 expression through a miRNA-dependent mechanism. **a**. RIP experiments were performed using the AGO2 antibody, and specific primers were used to detect the enrichment of HOXD-AS1 and SOX4. The data represent the average and standard deviation of three independent experiments. **b** Dicer deficiency rescued the siHOXD-AS1-mediated reduction of HOXD-AS1 and SOX4 mRNA in Huh7 cells. **c** Firefly luciferase activity normalized to Renilla luciferase activity in HEK-293 T cells co-transfected with luciferase reporters with wild type or mutant transcripts of HOXD-AS1 or SOX4 along with miR-130a-3p mimics or negative control (NC). **d** Protein levels of SOX4 following transfection of miR-130a-3p mimics or NC into Huh-7 and SMMC-7721 cells. **e** mRNA level of HOXD-AS1 was detected by biotin-coupled miR-130a-3p pull-down in Huh7 and SMMC-7721 cells. **f** Left: Protein level of SOX4 after transfection of miR-130a-3p mimics in SNU449 cells with HOXD-AS1 overexpression. Right: Protein level of SOX4 after transfection of miR-130a-3p inhibitors in Huh7 cells with HOXD-AS1 knockdown. Data in (**b**, **c**, **e**) are the means ± SD. *p < 0.05, **p < 0.01, and ***p < 0.001

Because HOXD-AS1 transcripts were abundant in the cytoplasm and the expression levels of HOXD-AS1 and SOX4 changed synchronously in HCC cells, we hypothesized that HOXD-AS1 may have the role as a ceRNA to regulate the expression of SOX4. To test this hypothesis, we first performed RNA immunoprecipitation (RIP) with an antibody against Argonaute (AGO2) in SMMC-7721 and Huh7 cells. We observed an enrichment of HOXD-AS1 and SOX4 with AGO2 antibody (Fig. 5a). These results indicated that HOXD-AS1 and SOX4 were recruited to AGO2 related RNA-induced silencing complexes (RISC) and might functionally interact with miRNAs. Additionally, we also evaluated the expression of HOXD-AS1 and SOX4 in Dicer-deficient Huh7 cells. We found that Dicer knockdown partially rescued the reduction of SOX4 by HOXD-AS1 knockdown (Fig. 5b). These results suggest that miRNAs may play essential roles in HOXD-AS1 mediated regulation of SOX4.

To investigate the potential miRNAs, we used bioinformatic tool miRanda to search for miRNAs that target the transcripts of HOXD-AS1 and SOX4. According to the prediction, miR-130a-3p, miR130b-3p, and miR454-3p have the same putative binding sites mapped to HOXD-AS1 and SOX4. We next analyzed expression status of three miRNAs in TCGA HCC cohort. The results showed that only miR-130a-3p was greatly reduced (Additional file 10: Figure S6A). In addition, previous study reported that miR-130a-3p could suppress cell migration and invasion in HCC cells [31]. These results suggest that miR-130a-3p may be a critical regulatory miRNA for HOXD-AS1 and SOX4.

To confirm HOXD-AS1 and SOX4 were regulated by miR-130a-3p, we constructed luciferase reporters containing wild type (WT) and mutated (MUT) putative binding sites of HOXD-AS1 or SOX4 transcripts, respectively (Additional file 10: Figure S6B-C). We found that reporter with wild type binding sites of HOXD-AS1 or SOX4 showed a markedly lower luciferase activity in HEK-293 T cells with overexpression of miR-130a-3p, however, we did not observe variations of luciferase activity in reporter with the mutated binding sites (Fig. 5c). To further confirm miRNAs-SOX4 interaction, protein level of SOX4 was evaluated in HCC cells transfected with miR-130a-3p. We found that miR-130a-3p suppressed endogenous SOX4 in HCC cells (Fig. 5d). Additionally, to validate the direct binding ability of miR-130a-3p on HOXD-AS1 and SOX4, we performed biotin-coupled miRNA pull down to capture HOXD-AS1 and SOX4 using streptavidin-coated beads from cells transfected with 3’end- biotinylated miR-130a-3p (Bi-has-miR-130a-3p). Control samples were transfected with a biotinylated C.elegans miRNA (Bi-cel-miR67). After 48 h transfection, we found that HOXD-AS1 was enriched to more than fourfold in Bi-miR-130a-3p pull down of HCC cells as compared to Bi-cel-miR67 transfection, and also we observed more than fivefold enrichment of SOX4 in the miR-130a-3p captured fraction compared with the negative control (Fig. 5e). Furthermore, we detected competitive binding activities of HOXD-AS1 and SOX4 to miR-130a-3p in SNU449 cells with HOXD-AS1 overexpression and Huh7 cells with HOXD-AS1 knockdown. Results showed that overexpression of HOXD-AS1 in SNU449 cells led to the decreased enrichment of SOX4 transcripts on miR-130a-3p. However, HOXD-AS1 knockdown in Huh7 cells caused a significant increase in the recruitment of SOX4 to miR-130a-3p (Additional file 11: Figure S7A-B).

To further explore the ceRNA role of HOXD-AS1, the expression levels of SOX4 after overexpression or knockdown of miR-130a-3p were examined. As shown in Fig. 5f, overexpression of miR-130a-3p could reduce the levels of SOX4, which rescued by the overexpression of HOXD-AS1. In addition, miR-130a-3p inhibitors nullified the inhibitory effect of HOXD-AS1 on the expression of SOX4 (Fig. 5f). These results demonstrate that HOXD-AS1 regulates SOX4 partly in miR-130a-3p dependent manner by sharing miRNAs response elements with SOX4.

### SOX4 is required for expression of MMP2 and EZH2 mediated by HOXD-AS1

**Fig. 6:**
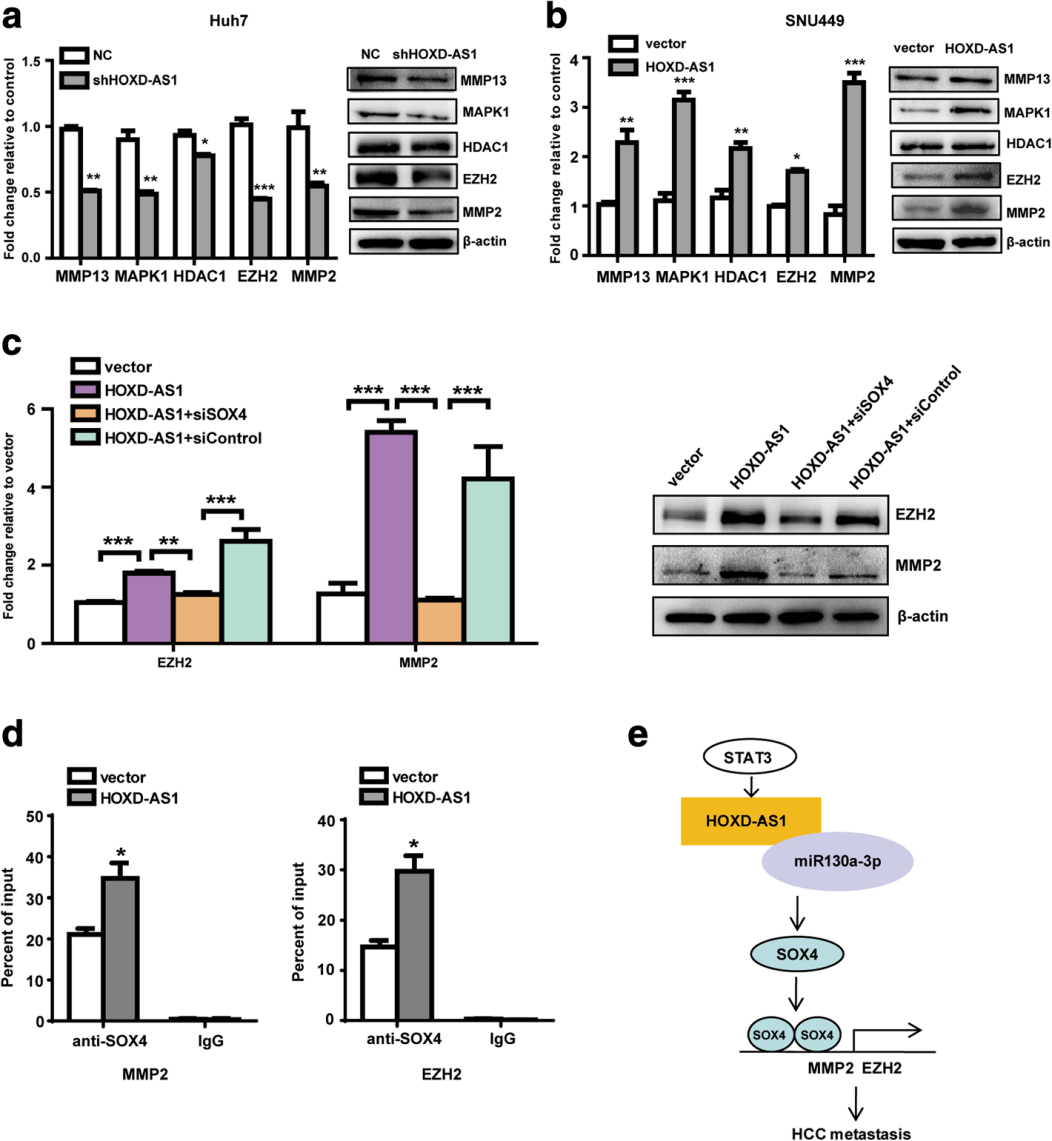
SOX4 is required for MMP2 and EZH2 expression mediated by HOXD-AS1. **a** mRNA and protein levels of metastasis-related genes in Huh7 cells with HOXD-AS1 knockdown. **b** mRNA and protein levels of metastasis-related genes in SNU449 cells with HOXD-AS1 overexpression. **c** The expression level change of EZH2 and MMP2 after knockdown of SOX4 in SNU449 cells with HOXD-AS1 overexpression. **d** ChIP assay was performed with antibody against SOX4 or control IgG in Huh28 cells with HOXD-AS1 overexpression or negative control. Immunoprecipitated DNA was analyzed by quantitative PCR. Genomic DNA input was 1%. **e** A proposed model for illustrating the function and mechanism of HOXD-AS1 in HCC metastasis. Data in A-D are the means ± SD. *p < 0.05, **p < 0.01, and ***p < 0.001

Because HOXD-AS1 promoted metastasis of HCC cells, it is important to explore whether some genes involved in this progression. We assessed the expression levels of 15 metastasis-related genes (Additional file 3: Table S3) in Huh7 cells with HOXD-AS1 knockdown. Our results showed that inhibition of HOXD-AS1 suppressed MMP13, MAPK1, HDAC1, EZH2, and MMP2 at both mRNA and protein levels compared with negative control (Fig. 6a). Conversely, overexpression of HOXD-AS1 increased mRNA and protein levels of these five genes in SNU449 cells (Fig. 6b). Next, we detected functional role of SOX4 on HOXD-AS1 regulation of these five genes. We found that knockdown of SOX4 strongly decreased expression levels of EZH2 and MMP2 that were enhanced by HOXD-AS1 overexpression (Fig. 6c), while the expression of MMP13, MAPK1, and HDAC1 were not affected (data not shown). Given that SOX4 is a transcription factor, we decided to investigate whether EZH2 and MMP2 is the target genes of SOX4. Chromatin immunoprecipitation (ChIP) with antibody against SOX4 or IgG was performed with Huh28 cell extracts of stable HOXD-AS1 overexpression or control. As expected, promoter regions of MMP2 and EZH2 were recruited to SOX4 in the control cells. Notably, the recruitment of the promoters of MMP2 and EZH2 to SOX4 was markedly increased in Huh28 cells with HOXD-AS1 overexpression (Fig. 6d). These results suggest that EZH2 and MMP2 are the direct target genes of SOX4, and HOXD-AS1 can mediate the expression of EZH2 and MMP2 through SOX4.

## Discussion

In the present study, we identified a number of lncRNAs that are aberrantly expressed in human HCC. Among them, HOXD-AS1 was most upregulated in HCC. High level of HOXD-AS1 expression was associated with significantly reduced overall survival, it may represent an independent prognostic biomarker in patients with HCC. LncRNAs with differential expression in cancer were correlated with good or bad prognosis, making them promising prognostic biomarkers [32]. For example, GAS5 was downregulated in several cancers [33–35], and low level of GAS5 indicated a poor prognosis in HCC [33]. Upregulation of HOTAIR was shown to be a marker of poor prognosis in a number of cancers, including HCC [36–41]. Moreover, increased HOTAIR was a prognostic biomarker of tumor recurrence following liver transplantation [42]. HOTTIP, HOXA13, and HEIH also act as independent prognostic factors associated with recurrence in HCC [43, 44], suggesting that lncRNAs have considerable prognostic potential in HCC.

Here, we identified the mechanism responsible for HOXD-AS1 upregulation in HCC cells. We first found that STAT3 could specific interact with the HOXD-AS1 promoter through the binding site located -938 nt ~ -928 nt and illustrated the mechanism by which STAT3 upregulated the expression of HOXD-AS1. The present results noticed that the region between -2000 nt to -746 nt on the HOXD-AS1 promoter contained regulatory elements for the transcription of HOXD-AS1. However, only the region between -1158 nt ~ -746 nt had the STAT3 binding site and contributed to mediate transcription of HOXD-AS1. This result indicates that there may be additional transcriptional factors in the region between -2000 to -1159 that regulate the transcription of HOXD-AS1.

It has been described that lncRNAs play an important role in hepatocarcinogenesis [45, 46]. In this study, we have shown that HOXD-AS1 can promote migration and invasion of HCC cells in vitro and contribute to distant lung metastasis in vivo. To our knowledge, this is the first study to report that HOXD-AS1 regulates cellular metastasis in HCC. In bladder cancer cells, knockdown of HOXD-AS1 could suppress cell proliferation and migration, and increased the rate of apoptotic cell [47]. Additionally, HOXD-AS1 was involved in angiogenesis and inflammation and controlled cell differentiation in neuroblastoma [48]. These findings further support the potential pro-oncogenic role of HOXD-AS1 in cancers. However, molecular mechanisms of HOXD-AS1 in pro-oncogenesis have not been clarified in human cancers, including HCC.

Based on global genome microarray data, we integrated gene co-expression patterns and identified positive correlation between HOXD-AS1 and SOX4 in HCC tissues. Synchronous change in expression levels of HOXD-AS1 and SOX4 was also detected in HCC cell lines. Previous studies reported that lncRNAs could crosstalk with protein-coding genes in a miRNA-dependent manner in HCC progression [13, 49, 50]. In this study, we revealed that HOXD-AS1 could function as a ceRNA that sponge miRNA130a-3p to protect SOX4 against degradation. Importantly, Liao et al. observed SOX4 potentiates metastasis in HCC [51]. The identification of a model of miRNA/lncRNA interaction may promote the understanding of the underlying mechanism of HCC metastasis.

We have also shown that 5 metastasis-related genes MMP13, MAPK1, HDAC1, EZH2, and MMP2 are required for HOXD-AS1 in the role of HCC metastasis. Among these five genes, we further found that EZH2 and MMP2 were the direct target genes of SOX4. Our results demonstrate that HOXD-AS1 is important for induction of SOX4, which result in enhanced expression of EZH2 and MMP2 and, in turn, lead to HCC metastasis. Additionally, the potential mechanism of MMP13, MAPK1, and HDAC1 regulated by HOXD-AS1 will be further analyze.

In conclusion, HOXD-AS1 is regulated by the transcriptional factor STAT3, it significantly upregulated in HCC and can be used as a prognosis biomarker for HCC patients. HOXD-AS1 functions as a ceRNA that competitively binds to miR-130a-3p, then upregulates SOX4 and promotes HCC cell metastasis. These findings provide a new mechanism for understanding HCC metastasis and HOXD-AS1 may be a potential candidate in the prevention and treatment of HCC.

## Conclusions

In this study, we reported that a number of lncRNAs are differentially expressed in HCC tissues. One of these lncRNAs, HOXD-AS1 was markedly upregulated in HCC. The high expression level of HOXD-AS1 was associated with poor prognosis and high tumor node metastasis stage in HCC patients and it may represent an independent prognostic biomarker in patients with HCC. Moreover, our results suggested that the transcription factor STAT3 could combine to the promoter of HOXD-AS1 and activate the transcription of HOXD-AS1. We further found that HOXD-AS1 facilitated HCC metastasis in vitro and in vivo. Furthermore, we had evidenced that HOXD-AS1 shared miRNA response elements with SOX4. Overexpression of HOXD-AS1 competitively bound to miR-130a-3p that prevented SOX4 from miRNA-mediated degradation, thus activated the expression of EZH2 and MMP2 and facilitated HCC metastasis. In conclusion: HOXD-AS1 is a prognostic marker for HCC patients and it may play a pro-metastatic role in hepatocarcinogenesis.

## Additional files

Additional file 1: Table S1. Clinicopathological characteristics of 14 HCC patients in global gene expression analysis. (DOCX 18 kb)

Additional file 2: Table S2. Oligonucleotide sequences in this study. (DOCX 19 kb)

Additional file 3: Table S3. Antibodies used in this study. (DOCX 15 kb)

Additional file 4: Table S4. The 26 differentially expressed lncRNAs in microarray analysis. (DOCX 18 kb)

Additional file 5: Figure S1. The fold change of HOXD-AS1 expression in 14 HCC tissues. (A) probe: 239182_a. (B) probe: 242042_s_at. (TIF 998 kb)

Additional file 6: Figure S2. RACE analysis of the full length of HOXD-AS1. (A) A garose gel electrophoresis of PCR products from 5’-RACE and 3’-RACE analysis of HOXD-AS1. (B) The nucleotide sequence of the full-length HOXD-AS1. (TIF 5631 kb)

Additional file 7: Figure S3. Analysis for HOXD-AS1 expression and phosphorylated STAT3 in HCC tissues. **(A)** The transcriptional expression level of HOXD-AS1 and phosphorylated STAT3 in 30 HCC tissues. **(B)** Correlation analysis between p-STAT3 (x) and HOXD-AS1 (y) in 30 HCC tissues (R = 0.53, p = 0.0023). (TIF 2920 kb)

Additional file 8: Figure S4. The expression level of HOXD-AS1 in HCC cells. (A) Real-time PCR analysis of HOXD-AS1 expression in 8 different HCC cell lines and normal liver cell L02. (B) Expression of HOXD-AS1 was quantified by Real-time PCR after knockdown of HOXD-AS1 in SMMC-7721 and Huh7 cells. (C) Expression levels of HOXD-AS1 in Huh28 and SNU449 cells that had been stably transfected with lentivirus encoding HOXD-AS1. (TIF 1314 kb)

Additional file 9: Figure S5. Expression correlation between HOXD-AS1 and transcription factors. (A) Expression correlation between HOXD-AS1 (x) and E2F7, E2F8, NFYA, and TCF19 (y) in cancerous tissues of the 14 HCC patients used in global gene expression analysis. Data are depicted as probe signal value of global gene expression microarray. (B) Real-time analysis for changes of E2F7, E2F8, NFYA, and TCF19 in Huh7 cells with HOXD-AS1 knockdown. (C) Real-time PCR analysis for changes of E2F7, E2F8, NFYA, and TCF19 in SNU449 cells with HOXD-AS1 overexpression. (TIF 1665 kb)

Additional file 10: Figure S6. Putative binding sites of HOXD-AS1 and SOX4 with miR-130a-3p. (A) Expression of potential miRNAs in HCC in TCGA cohorts. (B) Comparison summary of miR-130a-3p target sites in HOXD-AS1 and SOX4. The red nucleotides (target sites) were deleted in the mutant constructs. (C) pGL3 luciferase reporter constructs containing wild type and mutated putative binding sites of HOXD-AS1 or SOX4 transcripts were shown. (TIF 1598 kb)

Additional file 11: Figure S7. Competitive binding activities of HOXD-AS1 and SOX4 to miR-130a-3p. (A) Binding activities of HOXD-AS1 and SOX4 to miR-130a-3p in SNU449 cells with HOXD-AS1 overexpression. (B) Binding activities of HOXD-AS1 and SOX4 to miR-130a-3p in Huh7 cells with HOXD-AS1knockdown. (TIF 973 kb)

## Abbreviations

AGO2: Argonaute2
ceRNAs: Competing endogenous RNAs
ChIP: Chromatin immunoprecipitation
DMEM: Dulbecco’s modified eagle’s medium
E2F7: E2F transcription factor 7
E2F8: E2F transcription factor8
EZH2: Enhancer of zest homolog 2
FISH: Fluorescence in situ hybridization
GEO: Gene expression omnibus
HCC: Hepatocellular carcinoma
HDAC1: Histone deacetylase 1
lncRNA: Long noncoding RNA
MAPK1: Mitogen-activated protein kinase 1
miRNAs: MicroRNAs
MMP13: Matrix metallopeptidase 13
MMP2: Matrix metallopeptidase 2
NFYA: Nuclear transcription factor Y subunit alpha
RACE: Rapid amplification of cDNA ends
RIP: RNA immunoprecipitation
RISC: RNA-induced silencing complexes
shRNA: Short hairpin RNA
SOX4: SRY-box 4
STAT3: Signal transducer and activator of transcription 3
TCF19: Transcription factor 19
