# Supplementary material for: STAT3-mediated upregulation of lncRNA HOXD-AS1 as a ceRNA facilitates liver cancer metastasis by regulating SOX4
Source: Mol Cancer. 2017 Aug 14;16:136. doi: 10.1186/s12943-017-0680-1 (PMC5558651; doi:10.1186/s12943-017-0680-1)
Supplement: Supplementary file 1 — Clinicopathological characteristics of 14 HCC patients in global gene expression analysis. (DOCX 18 kb) [file 12943_2017_680_MOESM1_ESM.docx]

**Table S1. Clinicopathological characteristics of 14 HCC patients in global gene expression analysis**

| **Case** | **Sample Number** | **Age** | **Sex** | **HBsAg** | **tumor size** | **Distant**  **metastasis** | **Vascular invasion** | **Lymphatic**  **invasion** | **Cirrhosis** | **Pathologic diagnosis** | **Serum AFP**  **(ng/ml)** |
| --- | --- | --- | --- | --- | --- | --- | --- | --- | --- | --- | --- |
| G302 | S1-C/S2-N | 63 | Male | + | 7.3*6.6*6.5 | No | No | No | No | HCC | >400 |
| G304 | S3-C/S4-N | 35 | Male | + | 5*4*4 | No | No | No | No | HCC | 19.6 |
| G307 | S5-C/S6-N | 40 | Male | + | 9*8*8 | No | No | No | No | HCC,Ⅰ-Ⅱ | 520 |
| HK165 | S11-C/S12-N | 54 | Male | + | >10cm | No | Portal vein | Porta hepatic | Micronodular cirrhosis | HCC | 443.2 |
| G114 | S15-C/S16-N | 50 | Male | + | 7*9*7 | No | No | Yes | Micronodular cirrhosis | HCC | <10 |
| Q134 | S17-C/S18-N | 45 | Male | + | 3*2 | No | No | No | No | HCC, Ⅲ | >400 |
| Q142 | S21-C/S22-N | 43 | Female | + | 3*3*3 | No | Portal vein | No | Micronodular cirrhosis | HCC, Ⅲ | >400 |
| Q162 | S23-C/S24-N | 67 | Female | + | 10*12 at left lateral lobe and 3*2 at right lobe | Yes | Yes | Yes | Yes | HCC, II-III | 250 |
| D129 | S25-C/S26-N | 40 | Female | + | 20*15*18 | No | No | No | Micronodular cirrhosis | HCC, III | >1000 |
| Q135 | S27-C/S28-N | 44 | Female | - | 9*10*7 | No | No | No | No | HCC, II | <20 |
| G65 | S29-C/S30-N | 35 | Female | + | 11*10*4.5 | No | No | No | Micronodular cirrhosis | HCC, III | >400 |
| G320 | S31-C/S32-N | 54 | Male | + | 11*9*7 | No | No | No | No | HCC | (-) |
| HK113 | S33-C/S34-N | 50 | Male | + | ? | No | Portal vein | No | Mixed micro- and macronodular cirrhosis | HCC | 2047.1 |
| HK120 | S35-C/S36-N | ? | Male | ? | ? | ? | ? | ? | ? | HCC | ? |

Abbreviations: HCC, hepatocellular carinoma; HBsAg, hepatitis B surface antigen; AFP, α-fetoprotein; C, cancerous tissues; N, corresponding adjacent nontumorous tissues.
