# Supplementary material for: STAT3-mediated upregulation of lncRNA HOXD-AS1 as a ceRNA facilitates liver cancer metastasis by regulating SOX4
Source: Mol Cancer. 2017 Aug 14;16:136. doi: 10.1186/s12943-017-0680-1 (PMC5558651; doi:10.1186/s12943-017-0680-1)
Supplement: Supplementary file 2 — Oligonucleotide sequences in this study. (DOCX 19 kb) [file 12943_2017_680_MOESM2_ESM.docx]

**Table S2. Oligonucleotide sequences**

| **Gene** | **Sequence** |
| --- | --- |
| Primers for RACE |  |
| 3’-RACE GSP1 | 5’ CGGGCTGAATCTAAGCAGCCTGAC 3' |
| 3’-RACE GSP2 | 5’ GGTAAAATGTGCCTTCGATTGAAGTC 3’ |
| 5’-RACE GSP1 | 5’ CAGAGACACATCAGCCAAATTAG 3’ |
| 5’-RACE GSP2 | 5’ CTTCCCTAGTCTGTACCAGCC 3’ |
| HOXD-AS1 full length | F: 5' AGGCGCGCCAAATGGGAGCGTGACGCGCG 3' |
|  | R: 5' CCTTAATTAAAGACACTTTGAAAAAAATATTTTATTT 3' |
| HOXD-AS1 | F: 5’ CCTTGAAAGTGGGTAAAATGTGC 3’ |
|  | R: 5’ TAGTTTCCTTGTTCCTTTGTGCTGT 3’ |
| U1 snRNA | F: 5’ GGGAGATACCATGATCACGAAGGT 3’ |
|  | R: 5’ CCACAAATTATGCAGTCGAGTTTCCC 3’ |
| GAPDH | F: 5’ TGCACCACCAACTGCTTAGC 3’ |
|  | R:5’ GGCATGGACTGTGGTCATGAG 3’ |
| STAT3 | F: 5' CTCAACTTCAGACCCGTCAACA 3'  R: 5' GCTCCACGATTCTCTCCTCCA 3' |
| β-actin | F: 5’ AGTTGCGTTACACCCTTTCTTG 3’ |
|  | R: 5’ GCTGTCACCTTCACCGTTCC 3’ |
| Primers for TFs |  |
| E2F7 | F: 5’ TTCTAGCTCGCTATCCAAGTTATCC 3’ |
|  | R: 5’ CAGCGACTCCAGCACATTTACA 3’ |
| E2F8 | F: 5’ TCAACGCCTCAGATAGTAAGCC 3’ |
|  | R: 5’ GATAAGATCCAGGCTACTCAGAACA 3’ |
| SOX4 | F: 5’ GCAAACCAACAATGCCGAGAA 3’ |
|  | R: 5’ GCTTGATGTGCCCACTCGG 3’ |
| NFYA | F: 5’ CAGTGGCAGGCAATGTGGTC 3’ |
|  | R: 5’ CTCGGGCTTGCCTCCTCTTA 3’ |
| TCF19 | F: 5’ GCCTTCTGCTCCACCACAAC 3’ |
|  | R: 5’ GGGCTTTGTCTACACGGAGTTTC 3’ |
| Primers for metastasis-related genes | |
| MAPK1 | F: 5' GACCTCAAGCCTTCCAACCTG 3' |
|  | R: 5' TGGAGCCCTGTACCAACGTG 3' |
| HDAC1 | F: 5' CGAAGACGACCCTGACAAGC 3' |
|  | R: 5' ATCCTCTGTTTTGACTCTCTTGGC 3' |
| EZH2 | F: 5' TGGACCACAGTGTTACCAGCAT 3' |
|  | R: 5' GTGCTGGGCCTGCTACTGTTA 3' |
| TIMP2 | F: 5' CCTGGGACACCCTGAGCAC 3' |
|  | R: 5' ATCCAGAGGCACTCGTCCG 3' |
| MMP2 | F: 5' ATGCCGTCGTGGACCTGC 3' |
|  | R: 5' TGCTTCCAAACTTCACGCTCTT 3' |
| MMP3 | F: 5’ TGAGGACACCAGCATGAACC 3’ |
|  | R: 5’ ACTTCGGGATGCCAGGAAAG 3’ |
| MMP7 | F: 5' CGATGAGGATGAACGCTGGA 3' |
|  | R: 5' AGGAATGTCCCATACCCAAAGAA 3' |
| MMP9 | F: 5’GCCTTCGCACTGTGGAGC 3'  R: 5'GGATACCCGTCTCCGTGCTC 3' |
| MMP10 | F: 5' CACTGGAACCCTGAACCTGAAT 3'  R: 5' AATAAAAACGGTGTCCCTGCTG3' |
| MMP13 | F: 5' TCTTTCTTCGGCTTAGAGGTGACT 3' |
|  | R: 5' AAACATTGTATTCACCCACATCAGG 3' |
| MMP20 | F: 5' CTGAGAAGTGGACTATGGGAACG 3'  R: 5' TGCTGATGGGTCTGTGGAATG 3' |
| E-cadherin | F: 5' ACAATGCCGCCATCGCTTA 3'  R: 5' TGAACCACCAGGGTATACGTAGG3' |
| N-cadherin | F: 5’ GAGGCTTCTGGTGAAATCGC 3’  R: 5’ GCCACTTGCCACTTTTCCTG 3’ |
| TWIST1 | F: 5' CAAGAAGTCTGCGGGCTGTG 3'  R: 5' CCGCACGTTGGCCATGAC 3' |
| Nm23-H1 | F: 5' CAAGCGTTTTGAGCAGAAAGGAT 3'  R: 5'CGGCCCTGAGTGCATGTATTTC 3' |
| Primers for HOXD-AS1 promoter | |
| -2000bp~0bp | F: 5’ CCAAGTGAAATCCCTATTC 3’ |
|  | R: 5’ TAACCTTTTCCATGCCGCG 3’ |
| -745bp~0bp | F: 5’ CCAAGTGAAATCCCTATTC 3’ |
|  | R: 5’ AGCGTGGTGTCGCTGCCT 3’ |
| -324bp~0bp | F: 5’ CCAAGTGAAATCCCTATTC 3’  R: 5’ ACTGCTTGCACCTGAATG 3’ |
| -745bp~-325bp | F: 5’ TGGCTATCTCGATGCGCC 3’  R: 5’ AGCGTGGTGTCGCTGCCT 3’ |
| -1158bp~-746bp | F: 5’ CCCAGCGCATTTCCGCGG 3’  R: 5’ CGGACTACGGCTTCCTGG 3’ |
| -2000bp~-1159bp | F: CCAGGAAGCCGTAGTCCG 3’  R:5’ TAACCTTTTCCATGCCGCG 3’ |
| HOXD-AS1 FISH probe | 5’CTTGAGTGAAGTGCATCTTCTCAGCCTGCGGACC 3’ |
| SOX4 siRNA | 5’UGAAGCGCGUCUACCUGUUdTdT 3’ |
|  | 5’ AACAGGUAGACGCGCUUCAdTdT 3’ |
| STAT3 siRNA-1 | 5’GGGACCUGGUGUGAAUUAUdTdT 3’ |
|  | 5’AUAAUUCACACCAGGUCCCdTdT3’ |
| STAT3 siRNA-2 | 5’GGUACAUCAUGGGCUUUAU dTdT 3’ |
|  | 5’AUAAAGCCCAUGAUGUACC dTdT 3’ |
| siRNA control (NC) | 5’ UUCUCCGAACGUGUCACGUTT 3’ |
|  | 5’ ACGUGACACGUUCGGAGAATT 3’ |
| HOXD-AS1 shRNA | 5’GCAGAAGCCACAUUAAUAU 3’ |
| Dicer shRNA | 5’ AAGGGCACCCAUCUCUAAUUA 3’ |
| miR-130a-3p mimics | 5’ CAGUGCAAUGUUAAAAGGGCAU 3’ |
| mimics control  miR-130a-3p inhibitor | 5’ UCACAACCUCCUAGAAAGAGUAGA 3’  5’ AUGCCCUUUUAACAUUGCACUG 3’ |
| inhibitor control | 5’ UCUACUCUUUCUAGGAGGUUGUGA 3’ |
