# Supplementary material for: STAT3-mediated upregulation of lncRNA HOXD-AS1 as a ceRNA facilitates liver cancer metastasis by regulating SOX4
Source: Mol Cancer. 2017 Aug 14;16:136. doi: 10.1186/s12943-017-0680-1 (PMC5558651; doi:10.1186/s12943-017-0680-1)
Supplement: Supplementary file 3 — Antibodies used in this study. (DOCX 15 kb) [file 12943_2017_680_MOESM3_ESM.docx]

**Table S3. Antibodies used in this study**

| **Protein** | **Cat no.** | **Company** | **source** | **KD** |
| --- | --- | --- | --- | --- |
| SOX4 | ARP38234 | Aviva | Rabbit | 47 |
| AGO2 | sc-32877 | Santa cruz | Rabbit | 97 |
| MMP13 | 18165-1-AP | Protein tech | Rabbit | 54 |
| MAPK1 | 16443-1-AP | Protein tech | Rabbit | 41 |
| HDAC1 | [10197-1-AP](http://www.ptglab.com/Products/HDAC1-Antibody-10197-1-AP.htm) | Protein tech | Rabbit | 55 |
| STAT3 | [10253-2-AP](http://www.ptglab.com/Products/STAT3-Antibody-10253-2-AP.htm) | Protein tech | Rabbit | 86 |
| p-STAT3 | 94994 | CST | Rabbit | 86 |
| EZH2 | 21800-1-AP | Protein tech | Rabbit | 86 |
| MMP2 | [10373-2-AP](http://www.ptglab.com/Products/MMP2-Antibody-10373-2-AP.htm) | Protein tech | Rabbit | 72 |
| β-actin | A5441 | Sigma | Mouse | 42 |
