# Supplementary material for: STAT3-mediated upregulation of lncRNA HOXD-AS1 as a ceRNA facilitates liver cancer metastasis by regulating SOX4
Source: Mol Cancer. 2017 Aug 14;16:136. doi: 10.1186/s12943-017-0680-1 (PMC5558651; doi:10.1186/s12943-017-0680-1)
Supplement: Supplementary file 4 — The 26 differentially expressed lncRNAs in microarray analysis. (DOCX 18 kb) [file 12943_2017_680_MOESM4_ESM.docx]

**Table S4. The 26 differentially expressed lncRNAs in microarray analysis**

| **Probe Set ID** | **Chromosomal Location** | **Annotation Transcript Cluster** | **p-values** | **Fold change**  **(logC/N)** | **Up-regulation (C>N)** | **Down-regulation (C<N)** |
| --- | --- | --- | --- | --- | --- | --- |
| 1556185_a_at | chr7:87900208-87903065 | BC035072 | 0.0001 | -2.4785 | 0 | 14 |
| 1556316_s_at | chr22:24236604-24241118 | AI285192 | 0.0340 | 2.2708 | 12 | 2 |
| 1556913_a_at | chr12:110435224-110436932 | BC038793 | 0.0095 | 2.0331 | 10 | 4 |
| 1559528_at | chr4:773994-775636 | BC040652 | 0.0402 | 2.0572 | 12 | 2 |
| 1559573_at | chr4:185814153-185820506 | AK096134 | 0.0005 | -2.886 | 0 | 14 |
| 1563160_at | chr4:187187125-187187910 | BC020617 | 0.0184 | -2.1054 | 2 | 12 |
| 1563610_at | chr8:9182560-9192590 | AK055863 | 0.0039 | -2.4908 | 0 | 14 |
| 1566968_at | chr5:141697200-141697887 | AK024556 | 0.0022 | -2.274 | 2 | 12 |
| 1569433_at | chr6:147886863-147887919 | BC020896 | 0.004 | -2.0594 | 1 | 13 |
| 211456_x_at | chr1:237167402-237167718 | AF333388 | 0.0001 | -3.058 | 0 | 14 |
| 213929_at | chr11:108376161-108377780 | AL050204 | 0.0001 | -2.002 | 1 | 13 |
| 219791_s_at | chr4:174458465-174460620 | [NR_003679](http://genome.ucsc.edu/cgi-bin/hgc?hgsid=511171685_vejfziBvA4lU3zJANTYmlzlXIMC3&g=refGene&i=NR_003679&c=chr4&o=174451608&l=174451608&r=174462981&db=hg19) | 0.0235 | -2.7156 | 2 | 12 |
| 227404_s_at | chr5:137803735-137804994 | AI459194 | 0.0004 | -2.4811 | 0 | 14 |
| 228601_at | chr2:177037916-177039557 | AW340112 | 0.0135 | 3.0960 | 13 | 1 |
| 228977_at | chr13:21276265-21277151 | AI669535 | 0.0045 | 2.4648 | 13 | 1 |
| 230577_at | chr10:60759388-60761300 | AW014022 | 0.0009 | -3.8556 | 0 | 14 |
| 232090_at | chr1:172108191-172113708 | AI761578 | 0.0058 | -2.7179 | 2 | 12 |
| 232878_at | chr15:96812000-96870577 | AU143891 | 0.0013 | -2.3123 | 3 | 11 |
| 233604_at | chr3:108855560-108869074 | AI650260 | 0.0009 | -3.227 | 0 | 14 |
| 234139_s_at | chr1:57457022-57480189 | AK023382 | 0.003 | -2.6693 | 1 | 13 |
| 235891_at | chr1:172107542-172108270 | AI961147 | 0.0043 | -2.2481 | 3 | 11 |
| 236141_at | chr4:174461676-174463081 | AA156933 | 0.0092 | -2.4228 | 1 | 13 |
| 236652_at | chr20:42985164-42986109 | AW299927 | 0.0009 | -2.2731 | 1 | 13 |
| 238021_s_at | chr16:54952778-54962690 | AA954994 | 0.0063 | 2.8078 | 14 | 0 |
| 242881_x_at | chr14:19662801-19663445 | BG285837 | 0.0025 | 2.3743 | 14 | 0 |
| 244308_at | chr10:88964792-88965282 | BF514096 | 0.0048 | 2.4671 | 12 | 2 |
