# Supplementary figures and images for: STAT3-mediated upregulation of lncRNA HOXD-AS1 as a ceRNA facilitates liver cancer metastasis by regulating SOX4
Source: Mol Cancer. 2017 Aug 14;16:136. doi: 10.1186/s12943-017-0680-1 (PMC5558651; doi:10.1186/s12943-017-0680-1)

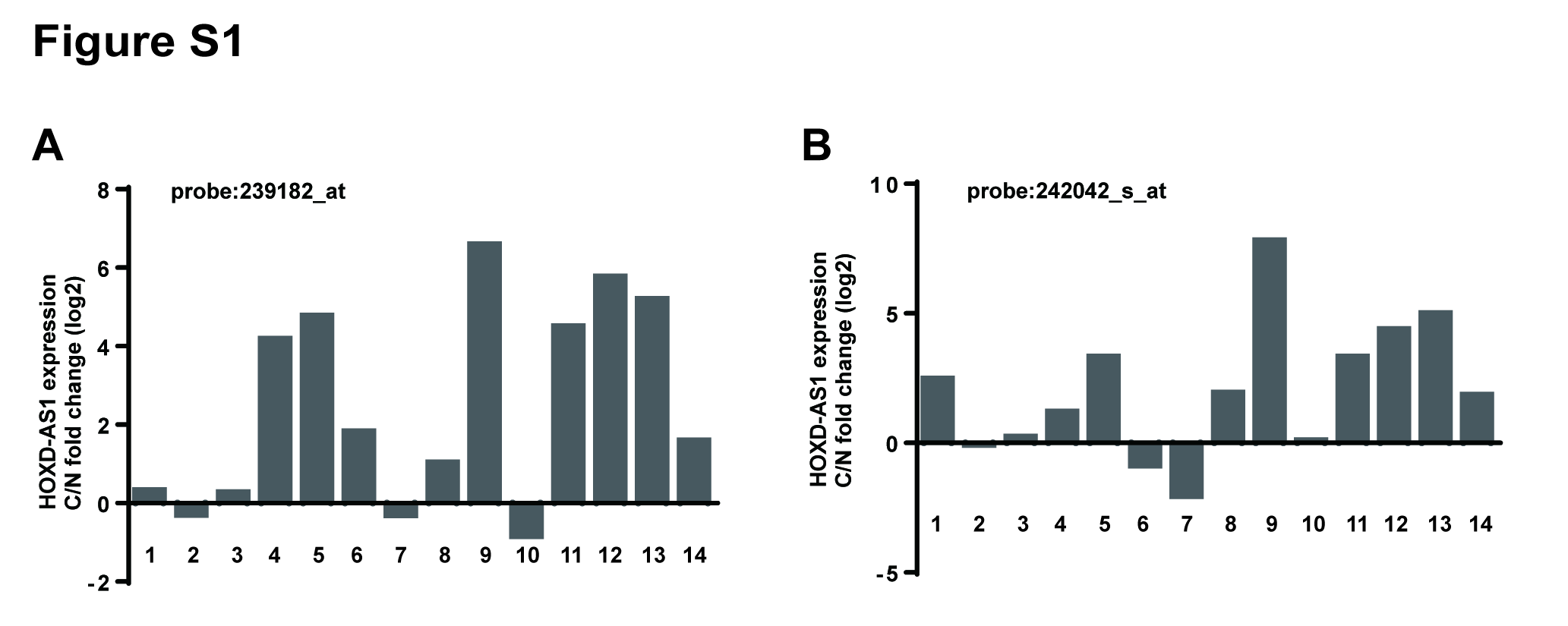

Supplement: Supplementary file 5 — The fold change of HOXD-AS1 expression in 14 HCC tissues. (A) probe: 239182_a. (B) probe: 242042_s_at. (TIF 998 kb) [file 12943_2017_680_MOESM5_ESM.tif]

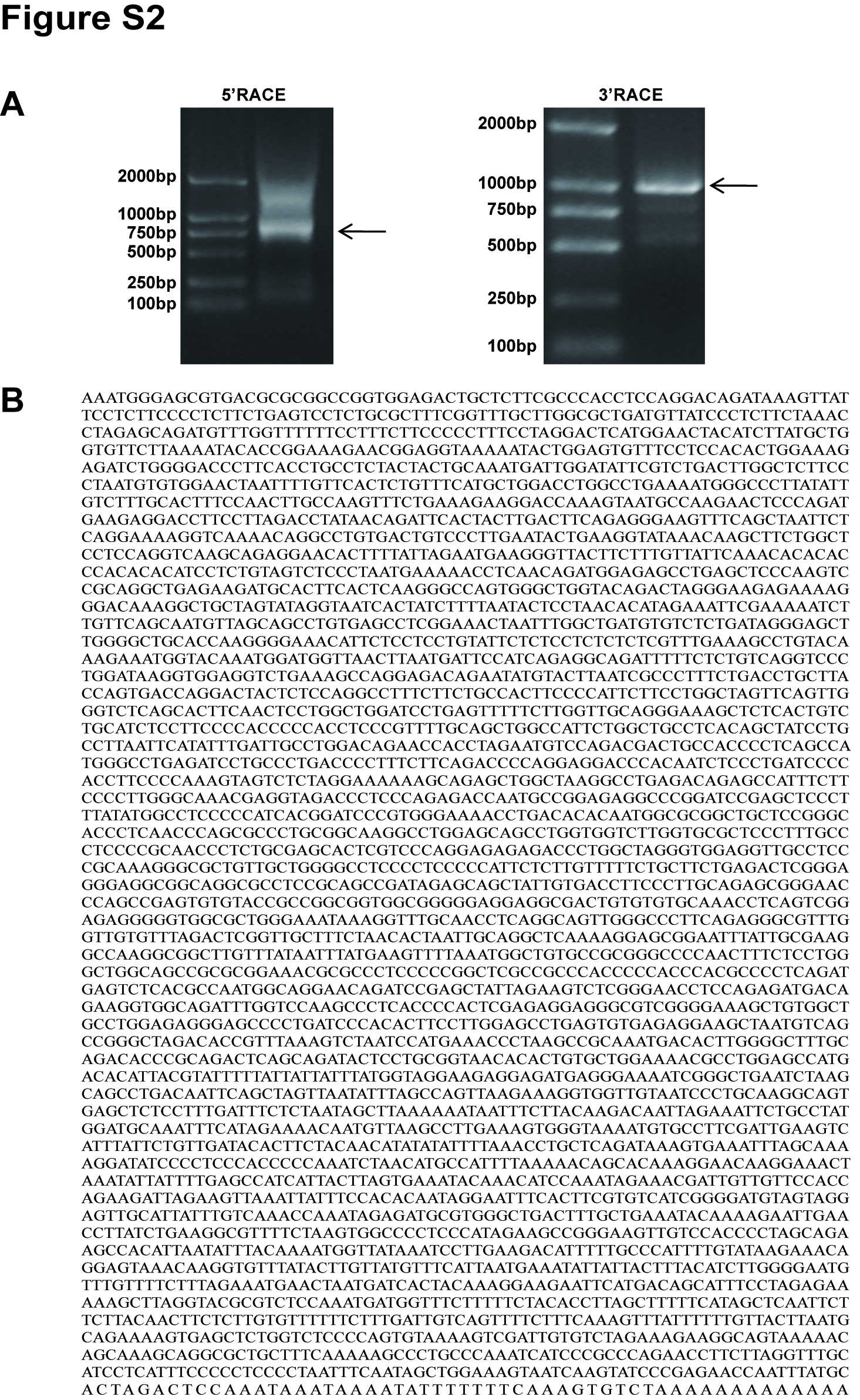

Supplement: Supplementary file 6 — RACE analysis of the full length of HOXD-AS1. (A) A garose gel electrophoresis of PCR products from 5’-RACE and 3’-RACE analysis of HOXD-AS1. (B) The nucleotide sequence of the full-length HOXD-AS1. (TIF 5631 kb) [file 12943_2017_680_MOESM6_ESM.tif]

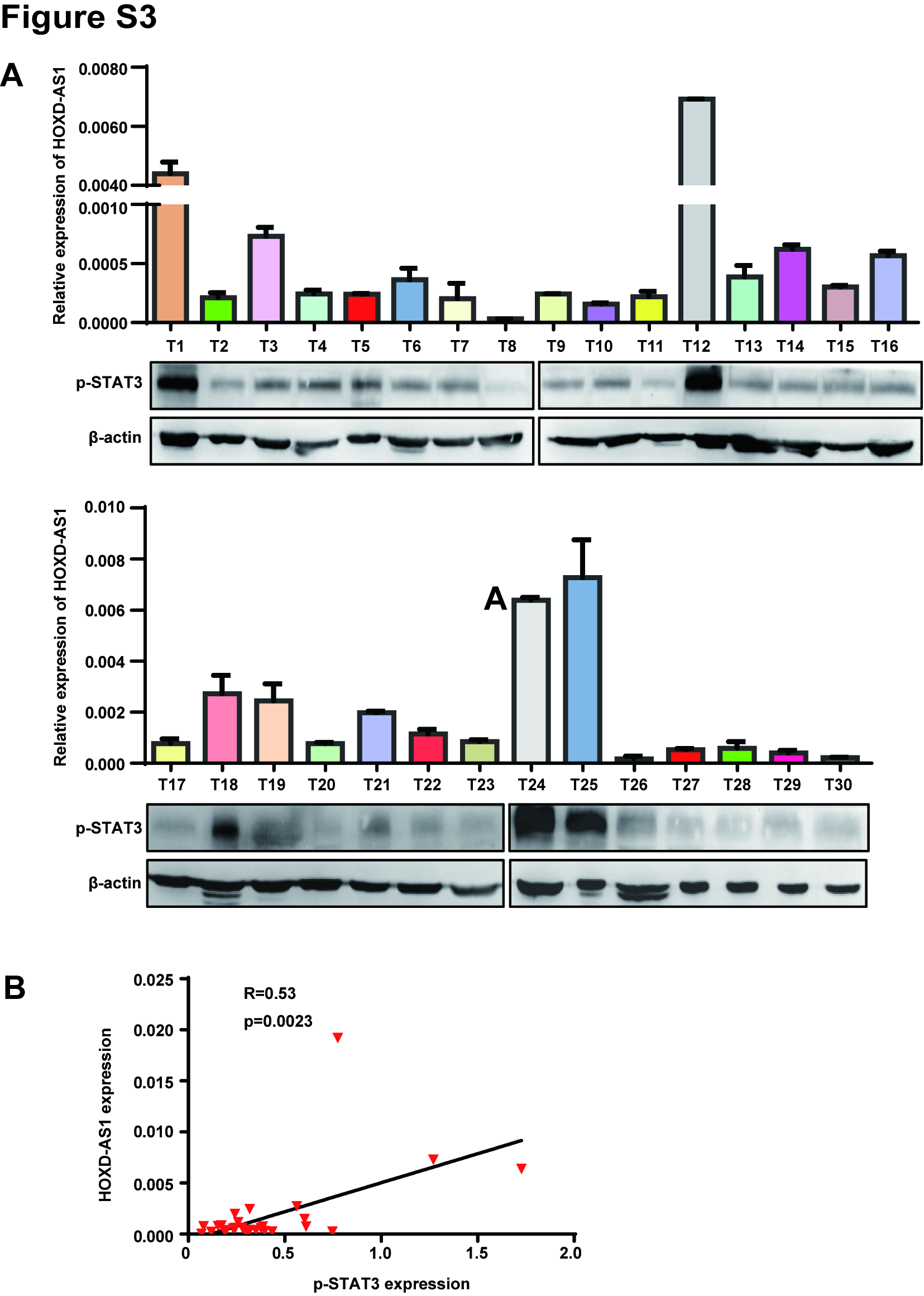

Supplement: Supplementary file 7 — Analysis for HOXD-AS1 expression and phosphorylated STAT3 in HCC tissues. (A) The transcriptional expression level of HOXD-AS1 and phosphorylated STAT3 in 30 HCC tissues. (B) Correlation analysis between p-STAT3 (x) and HOXD-AS1 (y) in 30 HCC tissues (R = 0.53, p = 0.0023). (TIF 2920 kb) [file 12943_2017_680_MOESM7_ESM.tif]

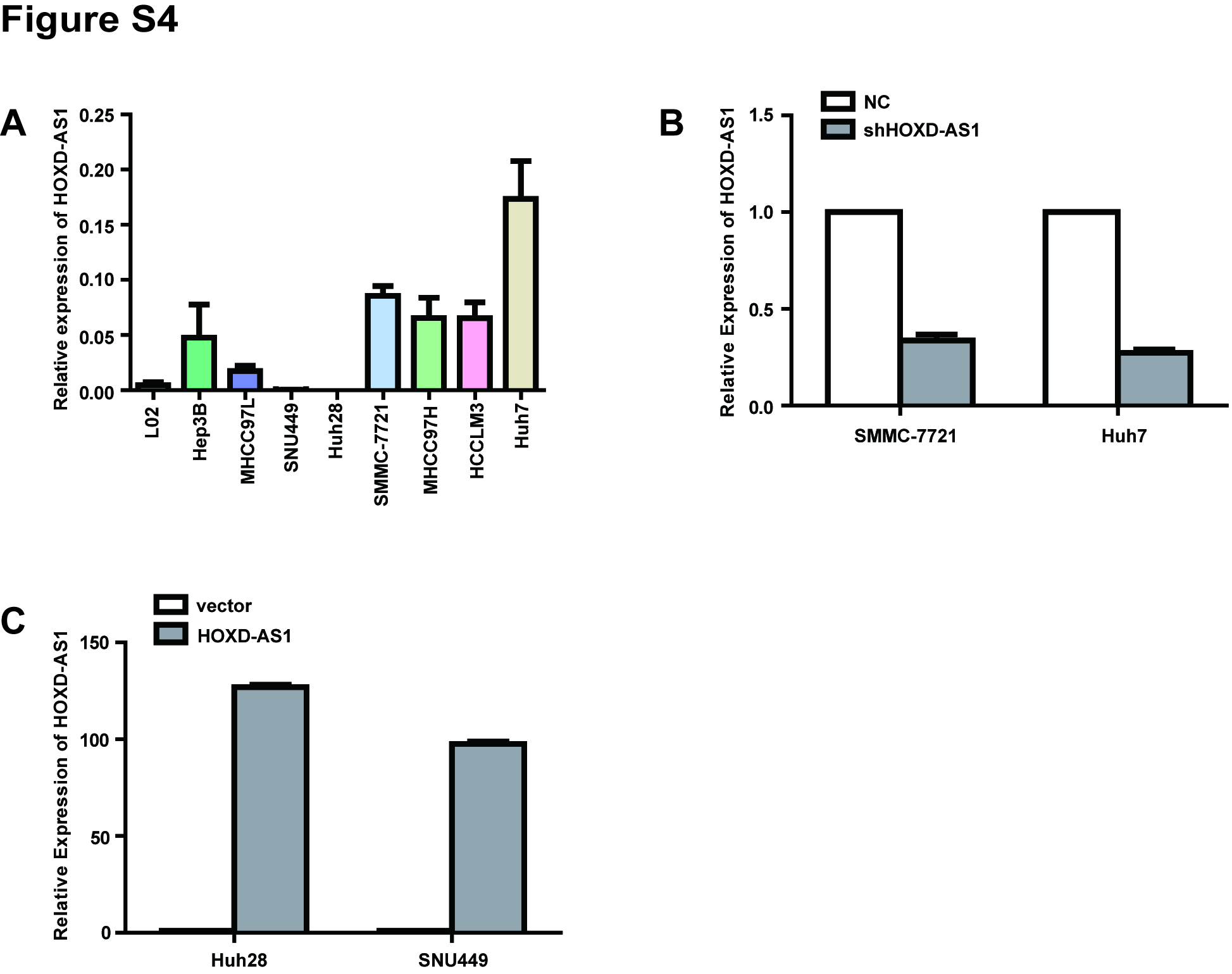

Supplement: Supplementary file 8 — The expression level of HOXD-AS1 in HCC cells. (A) Real-time PCR analysis of HOXD-AS1 expression in 8 different HCC cell lines and normal liver cell L02. (B) Expression of HOXD-AS1 was quantified by Real-time PCR after knockdown of HOXD-AS1 in SMMC-7721 and Huh7 cells. (C) Expression levels of HOXD-AS1 in Huh28 and SNU449 cells that had been stably transfected with lentivirus encoding HOXD-AS1. (TIF 1314 kb) [file 12943_2017_680_MOESM8_ESM.tif]

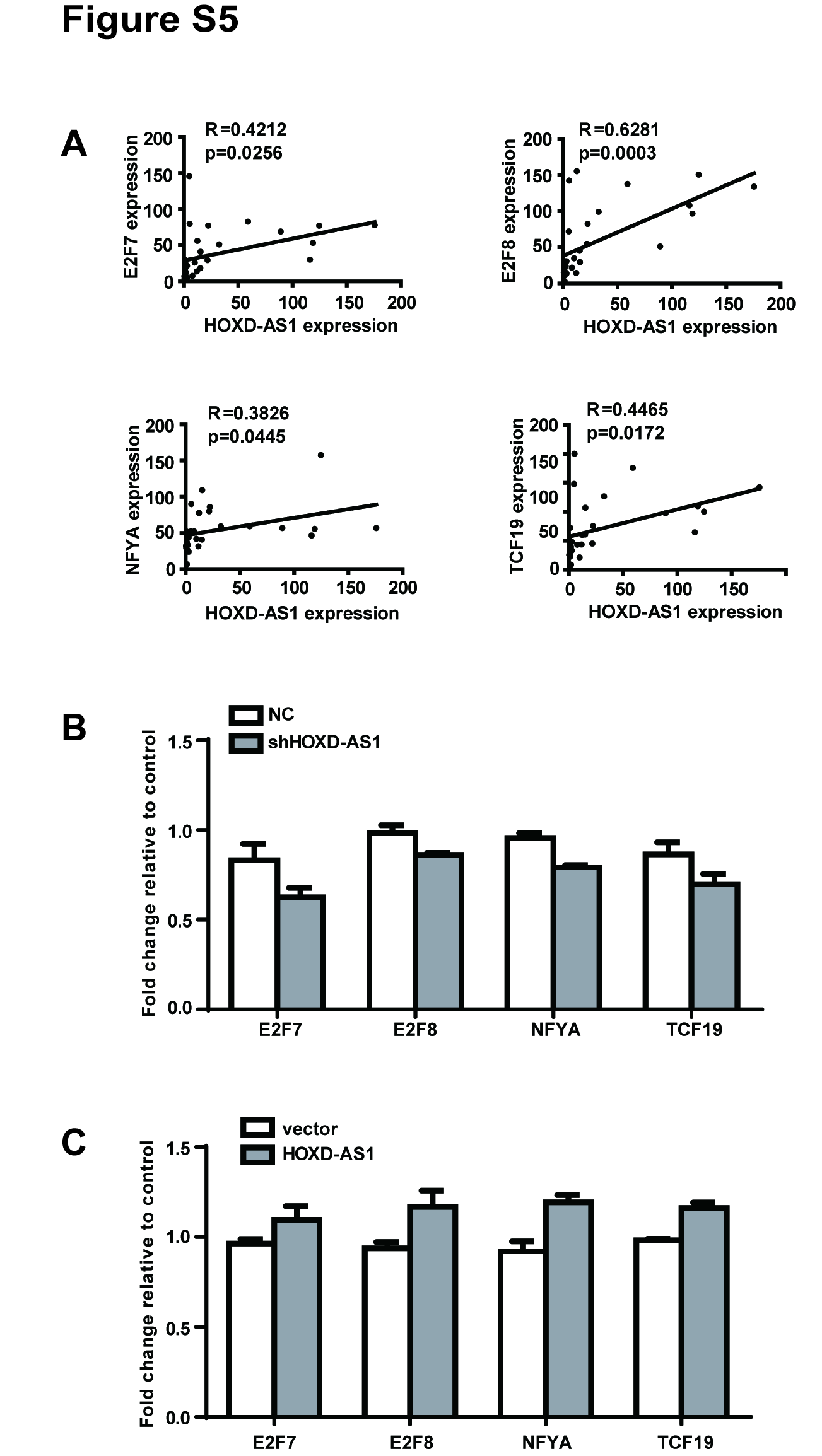

Supplement: Supplementary file 9 — Expression correlation between HOXD-AS1 and transcription factors. (A) Expression correlation between HOXD-AS1 (x) and E2F7, E2F8, NFYA, and TCF19 (y) in cancerous tissues of the 14 HCC patients used in global gene expression analysis. Data are depicted as probe signal value of global gene expression microarray. (B) Real-time analysis for changes of E2F7, E2F8, NFYA, and TCF19 in Huh7 cells with HOXD-AS1 knockdown. (C) Real-time PCR analysis for changes of E2F7, E2F8, NFYA, and TCF19 in SNU449 cells with HOXD-AS1 overexpression. (TIF 1665 kb) [file 12943_2017_680_MOESM9_ESM.tif]

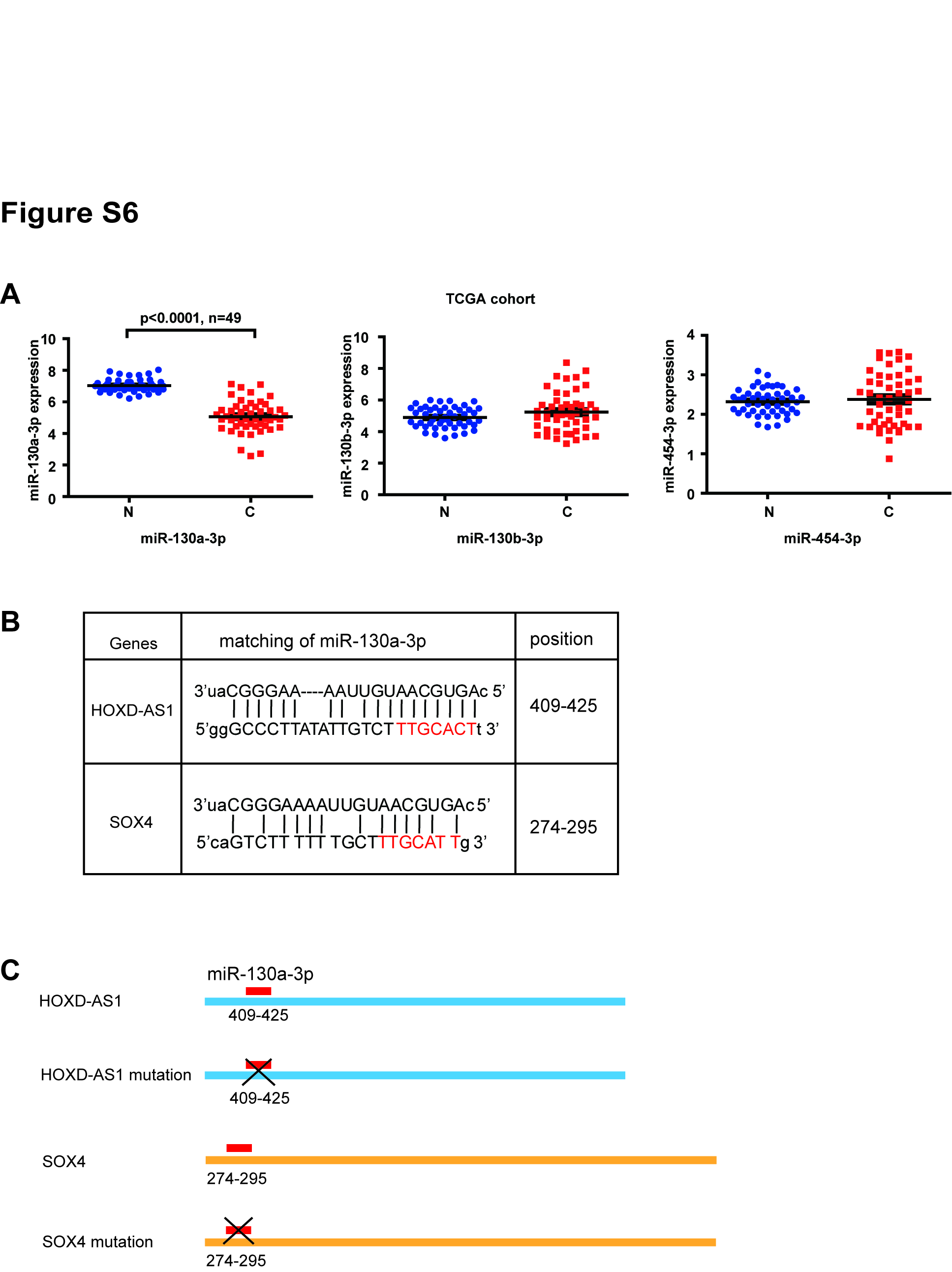

Supplement: Supplementary file 10 — Putative binding sites of HOXD-AS1 and SOX4 with miR-130a-3p. (A) Expression of potential miRNAs in HCC in TCGA cohorts. (B) Comparison summary of miR-130a-3p target sites in HOXD-AS1 and SOX4. The red nucleotides (target sites) were deleted in the mutant constructs. (C) pGL3 luciferase reporter constructs containing wild type and mutated putative binding sites of HOXD-AS1 or SOX4 transcripts were shown. (TIF 1598 kb) [file 12943_2017_680_MOESM10_ESM.tif]

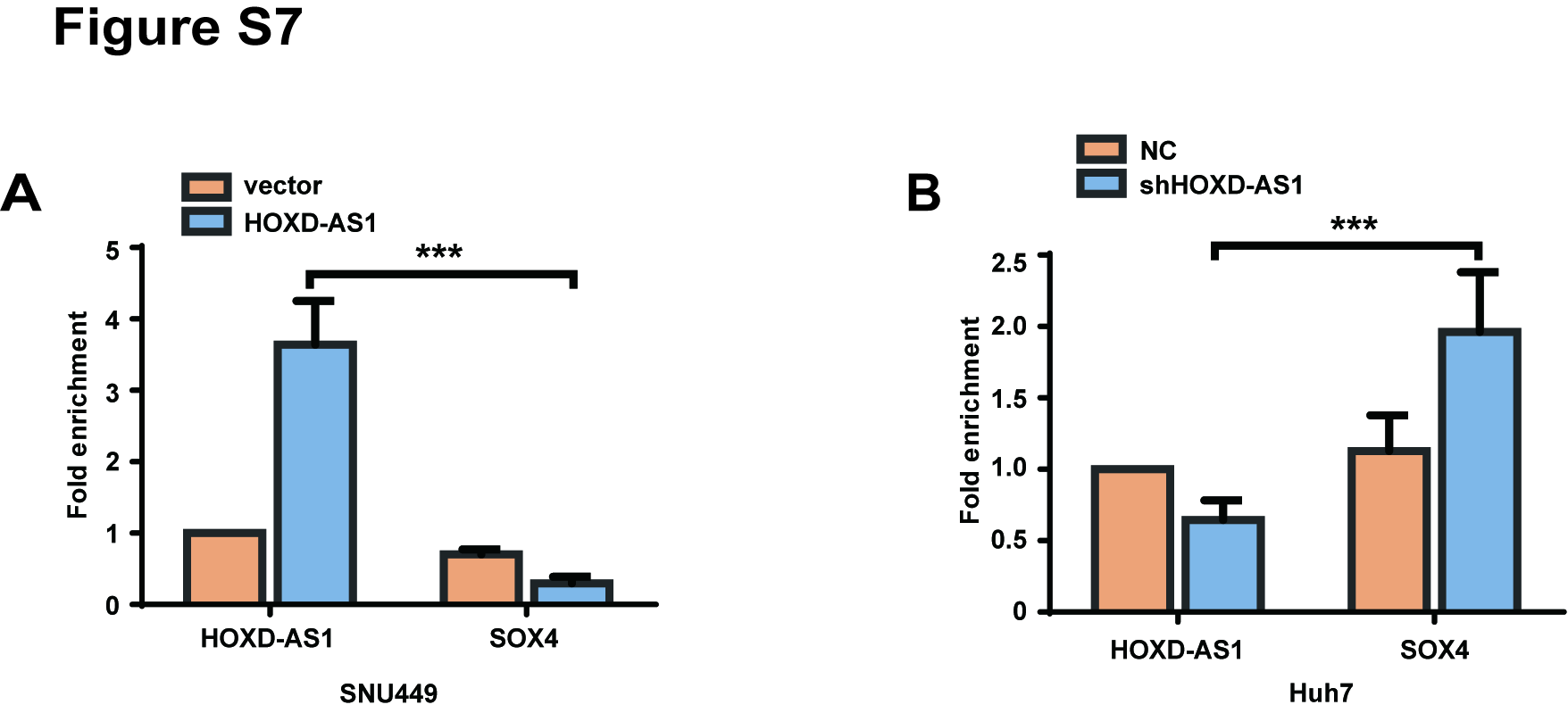

Supplement: Supplementary file 11 — Competitive binding activities of HOXD-AS1 and SOX4 to miR-130a-3p. (A) Binding activities of HOXD-AS1 and SOX4 to miR-130a-3p in SNU449 cells with HOXD-AS1 overexpression. (B) Binding activities of HOXD-AS1 and SOX4 to miR-130a-3p in Huh7 cells with HOXD-AS1knockdown. (TIF 973 kb) [file 12943_2017_680_MOESM11_ESM.tif]
